# LRP1 regulates asthmatic airway smooth muscle proliferation through FGF2/ERK signaling

**DOI:** 10.1172/jci.insight.185975

**Published:** 2025-05-08

**Authors:** Ya Deng, Jiaying Zhao, Chen Gong, Wenqian Ding, Lulu Fang, Huaqing Liu, Ming Li, Bing Shen, Shenggang Ding

**Affiliations:** 1 Department of Pediatrics, The First Affiliated Hospital of Anhui Medical University, Hefei, China.; 2 Department of Pediatrics, The Second Affiliated Hospital of Anhui Medical University, Hefei, China.; 3 Department of Pediatrics, East District of Hefei Maternal and Child Health Hospital, Hefei, China.; 4 Dr. Neher’s Biophysics Laboratory for Innovative Drug Discovery, State Key Laboratory of Quality Research in Chinese Medicine, Macau University of Science and Technology, Macau, China.; 5 School of Basic Medical Sciences, Anhui Medical University, Hefei, China.; 6 Beijing Children’s Hospital, Capital Medical University, China National Clinical Research Center of Respiratory Diseases, Beijing, China.

**Keywords:** Cell biology, Pulmonology, Asthma

## Abstract

Airway smooth muscle (ASM) hyperplasia is a hallmark of airway remodeling in asthma, which still lacks an effective treatment. Low-density lipoprotein receptor-related protein 1 (LRP1) is involved in regulating the proliferation of various cell types, and the intracellular domain of LRP1 (LRP1-ICD) also exhibits unique biological functions. However, the role of LRP1 in asthma airway remodeling remains unclear. In the present study, LRP1 was increased in ASM cells of mice with OVA-induced chronic asthma, with the elevation in LRP1-ICD protein levels being significantly greater than that of the LRP1 β chain. In vivo experiments demonstrated that inhibiting LRP1 reduced ASM proliferation in these mice. Mechanistically, *LRP1* knockdown inhibited the FGF2/ERK signaling pathway, thereby arresting cell cycle progression and suppressing ASM cell proliferation. Additionally, in vitro experiments revealed that the inhibitory effect of LRP1-ICD overexpression on ASM cell proliferation was lost after adjusting the levels of LRP1. LRP1-ICD overexpression inhibited full-length LRP1 protein levels by promoting its protein degradation rather than by suppressing its transcription, thus preventing further exacerbation of asthma. In conclusion, this study clarifies the molecular biological mechanism by which LRP1 regulates ASM proliferation, suggesting targeting full-length LRP1 as a strategy for therapeutic intervention in asthma airway remodeling.

## Abstract 2

This study reveals how LRP1 regulates airway smooth muscle proliferation, offering a novel potential target for preventing and treating airway remodeling in asthma.

## Introduction

Asthma is a major public health issue worldwide, affecting over 300 million people globally (1, 2). It is a complicated and highly heterogeneous disease that can cause recurrent episodes of coughing, wheezing, shortness of breath, and chest tightness, adversely affecting the quality of life for both children and adults (3). A typical feature of asthma is airway obstruction caused by the narrowing of the airway lumen. In the majority of cases, this airway narrowing can be reversed with bronchodilator medication. However, in certain severe forms of asthma, airway obstruction does not always normalize with routine therapy. An important cause of persistent airflow limitation in these patients is airway remodeling (4). Airway remodeling is a concise term that encompasses airway smooth muscle (ASM) proliferation, goblet cell metaplasia, excessive subepithelial collagen deposition, and increased vascularization. In 1922, Huber and Koesler first reported an increase in ASM tissue in people with asthma (5). Since then, ASM proliferation has garnered considerable attention as a histopathologic hallmark of asthma (6–8). It has been found that abnormal proliferation of ASM correlates with the severity of asthma and is a reason for reduced lung function (9). However, effective methods to prevent and treat asthma-associated ASM remodeling are still lacking.

Low-density lipoprotein receptor-related protein 1 (LRP1) is a ubiquitously expressed cell surface receptor that plays a multifaceted role in controlling inflammation, tissue remodeling, extracellular molecule clearance, and cell signal transduction (10–14). The *LRP1* gene can produce only one transcript upon transcription, which is subsequently translated to the LRP1 precursor protein. The LRP1 precursor protein, after the removal of signal peptide sequence, is processed into the LRP1 mature peptide. The LRP1 mature peptide undergoes posttranslational modifications to form a 515 kDa α chain and an 85 kDa β chain, which associate noncovalently at the cell membrane (15). Due to the need for protein denaturation before immunoblotting experiments, noncovalent bonds are broken. Consequently, the association between the LRP1 α chain and β chain is disrupted, forming free subunits. The large molecular weight of the α chain makes it relatively difficult to detect in conventional immunoblotting experiments. Therefore, in most previous LRP1-related studies, the protein levels of full-length LRP1 are primarily reflected by measuring the relative content of the β chain (12, 16–18). Membrane type-1 matrix metalloproteinase (MT1-MMP) (19, 20) or a disintegrin and a metalloproteinase 17 (ADAM17) (21) proteolytically processes LRP1, leading to shedding of the extracellular domain of LRP1. Subsequently, γ-secretase further hydrolyzes LRP1 on the inner side of the cell membrane to produce the intracellular domain of LRP1 (LRP1-ICD), a process that occurs constitutively in mammalian cells (22, 23). May et al. previously showed that the shedding of the extracellular domain is the rate-limiting step for the generation of LRP1-ICD (22). In a later study, Zurhove et al. proposed that LRP1-ICD, released from the plasma membrane, can translocate to the nucleus to regulate the transcription of inflammation-related genes (24). A previous GWAS identified SNPs within the *LRP1* gene that are associated with lung function traits (25). However, the role of LRP1, especially LRP1-ICD, in asthma airway remodeling has not been investigated.

In the current study, we first identified an upregulation of LRP1 in airway tissues of mice with OVA-induced chronic asthma and found a significantly greater increase in LRP1-ICD protein levels compared with the LRP1 β chain. We then knocked down *LRP1* and overexpressed LRP1-ICD in vitro to separately observe the effects of full-length LRP1 and LRP1-ICD on the function of human bronchial smooth muscle cells (HBSMCs). Finally, we verified the in vitro results using in vivo experiments. Overall, our results elucidated the mechanism underlying the role of LRP1 in ASM cell proliferation and provided a target for the clinical treatment of airway remodeling in asthma.

## Results

### Upregulation of LRP1 expression in ASM cells of mice modeling chronic asthma induced by OVA.

**Figure 1:**
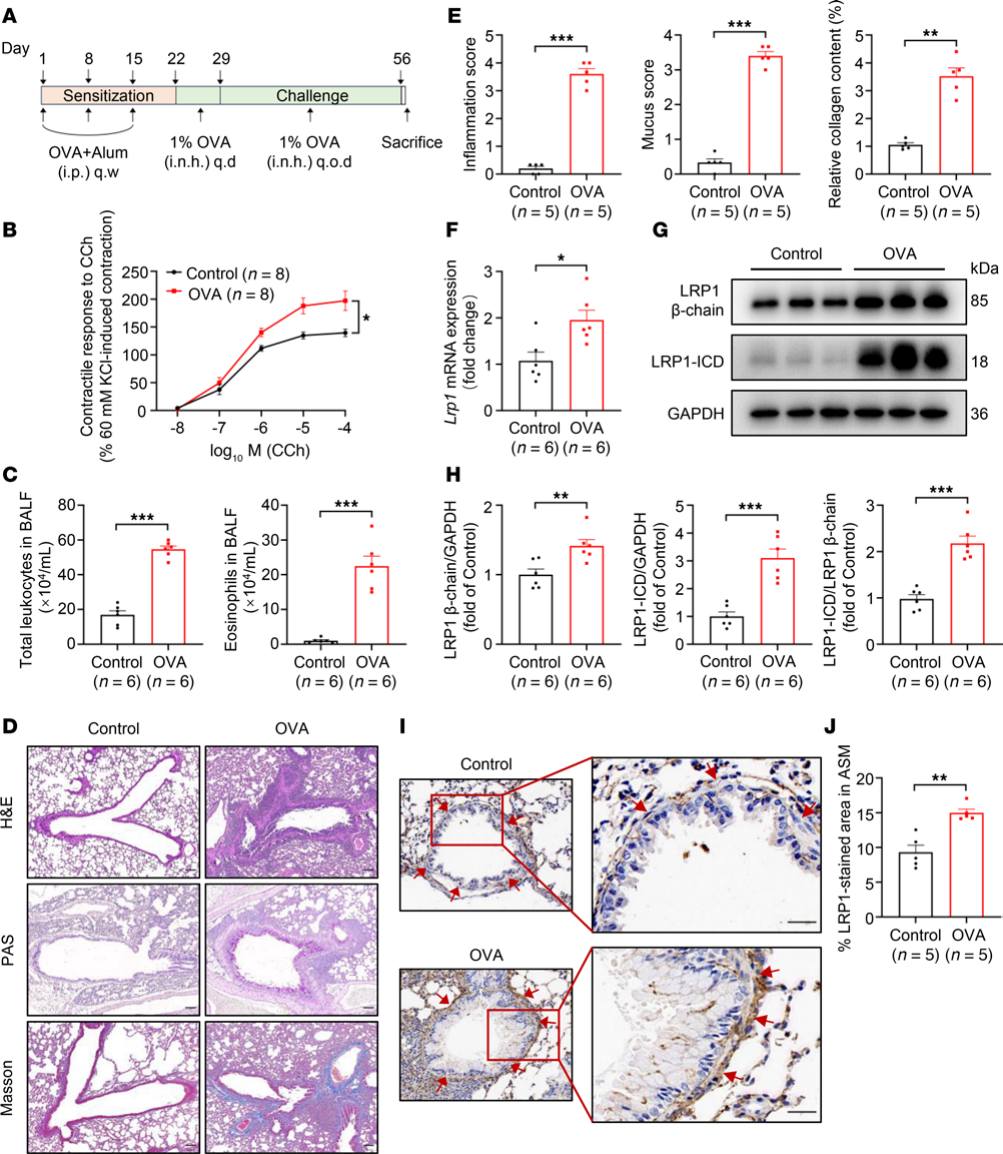
Upregulation of LRP1 expression in ASM cells of mice modeling chronic asthma induced by OVA. (**A**) Timeline for the construction of the OVA-induced chronic asthma mouse model. The mice were assigned to 1 of 2 groups: control and OVA-induced chronic asthma (OVA). i.p., intraperitoneal; q.w., once a week; i.n.h., nebulized inhalation; q.d., once a day; q.o.d., every other day. (**B**) Statistical curves showing contractile responses of tracheal rings to cumulative concentrations of carbachol (CCh) (10^–8^ to 10^–4^ mol/L). *n* = 8 tracheal rings from 4 mice per group. (**C**) Summary data showing the total leukocyte count and eosinophil count in the bronchoalveolar lavage fluid (BALF) of indicated mice. *n* = 6 mice per group. (**D**) Representative images of hematoxylin and eosin (H&E) staining, periodic acid–Schiff (PAS) staining, and Masson’s staining of lung tissues from both groups of mice. Scale bar: 100 μm. (**E**) Summary data showing inflammation score, mucus score, and relative collagen content of lung tissues in both groups of mice. *n* = 5 mice per group. (**F**) Summary data showing the relative expression levels of *Lrp1* mRNA in tracheal tissues of both mouse groups. *GAPDH* was used as an internal reference. *n* = 6 mice per group. (**G** and **H**) Representative immunoblot images (**G**) and summary data (**H**) showing LRP1 protein levels in tracheal tissues of indicated mice. *n* = 6 mice per group. (**I** and **J**) Representative immunohistochemistry images (**I**) and summary data (**J**) showing LRP1 protein levels in ASM of indicated mice. Red arrows indicate the ASM cells. Scale bar: 25 μm. *n* = 5 mice per group. All data were analyzed using independent-sample *t* tests or 2-way ANOVA and are presented as means ± SEMs. *P < 0.05, **P < 0.01, ***P < 0.001.

To explore the differential expression of LRP1 in airway tissues in asthma, we established an OVA-induced mouse model of chronic asthma (Figure 1A). Ex vivo tracheal tension measurements revealed that tracheal rings derived from mice in the OVA group showed a significantly enhanced contractile response to carbachol compared with mice in the control group (Figure 1B). Additionally, total leukocyte counts and eosinophil counts were significantly higher in the bronchoalveolar lavage fluid (BALF) of mice in the OVA group compared with control mice (Figure 1C). Histological analysis showed that, compared with control mice, OVA-treated mice exhibited increased peribronchial inflammatory infiltration, goblet cell hyperplasia in the bronchial epithelium, and increased peribronchial collagen deposition (Figure 1, D and E). These results indicate that the OVA-induced chronic asthma mouse model was successfully constructed.

Quantitative real-time PCR (qRT-PCR), Western blotting, and immunohistochemistry analyses were used to detect the expression levels of LRP1 in the airway tissues of the 2 groups of mice. The *Lrp1* mRNA and LRP1 protein levels in the tracheal tissues of mice modeling asthma were significantly higher than those in control mice (Figure 1, F–H). Interestingly, the increase in LRP1-ICD protein levels was significantly higher than that of the LRP1 β chain (Figure 1, G and H). However, there was no significant difference in LRP1 protein expression levels in the lung tissues between the 2 groups of mice (Supplemental Figure 1, A and B; supplemental material available online with this article; https://doi.org/10.1172/jci.insight.185975DS1). Immunohistochemical analysis of lung tissue showed significantly elevated LRP1 protein expression in the ASM cells of mice in the OVA group compared with control mice (Figure 1, I and J). Based on these findings, we conducted a series of in vitro studies using HBSMCs to investigate the function of LRP1 and the mechanism underlying that function.

### Effects of LRP1 knockdown on HBSMC function.

**Figure 2:**
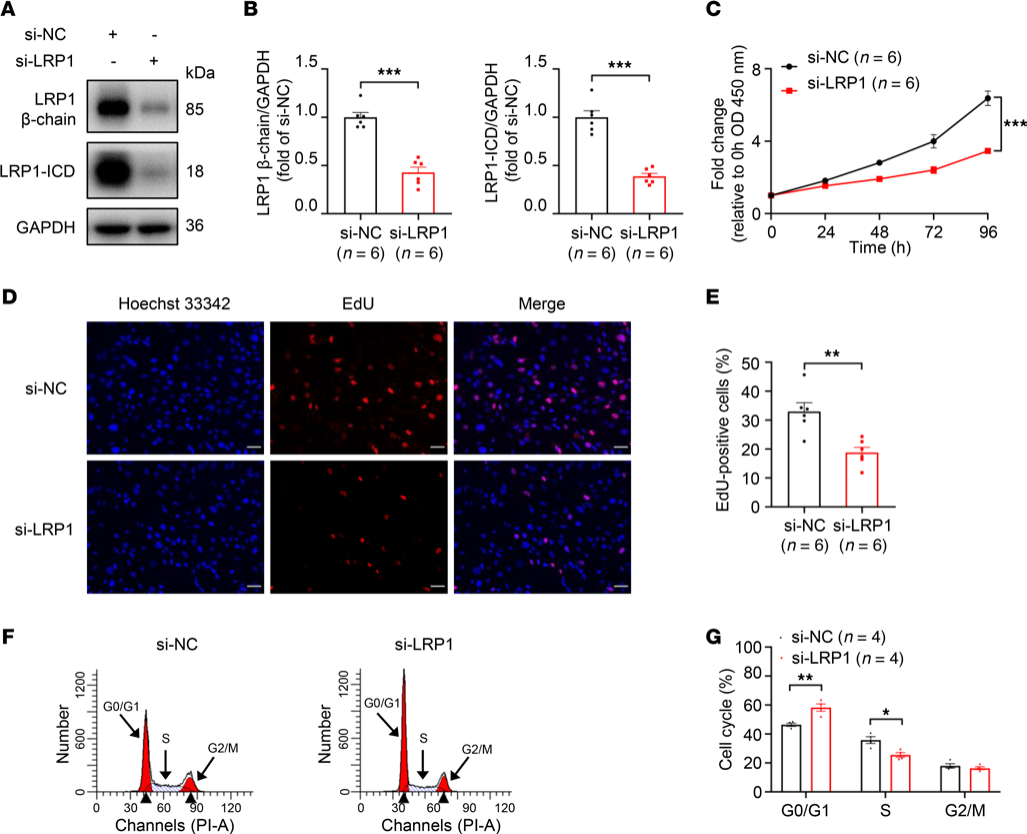
Effects of *LRP1* knockdown on HBSMC function. (**A** and **B**) HBSMCs were transfected with *LRP1*-targeting siRNA (si-LRP1) or negative control siRNA (si-NC). Representative immunoblot images (**A**) and summary data (**B**) showing LRP1 protein levels (*n* = 6). (**C**) Statistical curves from the CCK-8 assay showing the cell proliferation rate (*n* = 6). (**D** and **E**) Representative fluorescence images (**D**) and summary data (**E**) from the EdU incorporation assay showing the proportion of proliferating cells (*n* = 6). Scale bar = 50 μm. (**F** and **G**) Representative histograms (**F**) and summary data (**G**) showing the proportion of cells in G0/G1, S, and G2/M phases (*n* = 4). PI-A, propidium iodide area. All data were analyzed using independent-sample *t* tests or 2-way ANOVA and are presented as means ± SEMs. *P < 0.05, **P < 0.01, ***P < 0.001.

To investigate the function of LRP1 in HBSMCs, we specifically knocked down *LRP1* using an *LRP1*-targeting siRNA. The transfection efficiency was validated by Western blotting. As shown in Figure 2, A and B, the protein levels of the β chain and ICD were both significantly reduced, with a knockdown efficiency of more than 50%. Cell proliferation was assessed using the Cell Counting Kit-8 (CCK-8) assay (Figure 2C) and a 5-ethynyl-2′-deoxyuridine (EdU) incorporation assay (Figure 2, D and E). The results suggested that cell proliferation was significantly reduced in *LRP1* siRNA-transfected HBSMCs compared with the negative control siRNA–transfected (NC siRNA–transfected) HBSMCs. Flow cytometry was used to detect cell cycle and apoptosis. Compared with the NC siRNA transfection in HBSMCs, transfection with *LRP1*-specific siRNA significantly increased the proportion of cells in the G0/G1 phase and decreased the proportion of cells in the S phase (Figure 2, F and G). This finding indicated that *LRP1* knockdown caused cell cycle arrest at the G0/G1 phase and inhibited the transition from G1 to S phase. However, there was no significant difference in the number of apoptotic cells between the NC siRNA– and *LRP1* siRNA–transfected HBSMCs (Supplemental Figure 2, A and B). These results suggest that knockdown of *LRP1* may affect cell proliferation by altering cell cycle distribution rather than through apoptosis.

### Effects of LRP1-ICD overexpression on HBSMC function.

**Figure 3:**
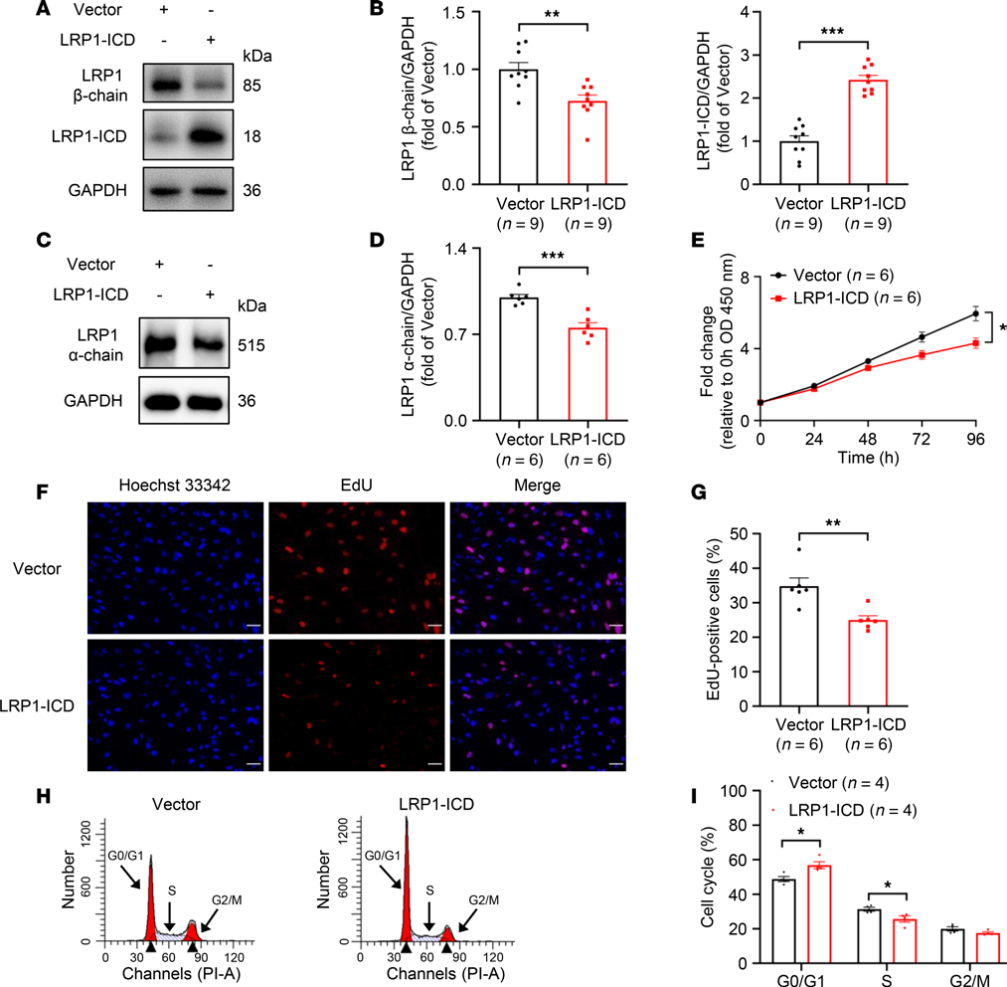
Effects of LRP1-ICD overexpression on HBSMC function. (**A** and **B**) HBSMCs were transfected with recombinant plasmids overexpressing LRP1-ICD (LRP1-ICD) or empty vector (Vector). Representative immunoblot images (**A**) and summary data (**B**) showing LRP1 β chain and LRP1-ICD protein levels (*n* = 9). (**C** and **D**) Representative immunoblot images (**C**) and summary data (**D**) showing LRP1 α chain protein levels (*n* = 6). (**E**) Statistical curves from the CCK-8 assay showing the cell proliferation rate (*n* = 6). (**F** and **G**) Representative fluorescence images (**F**) and summary data (**G**) from the EdU incorporation assay showing the proportion of proliferating cells (*n* = 6). Scale bar = 50 μm. (**H** and **I**) Representative histograms (**H**) and summary data (**I**) showing the proportion of cells in G0/G1, S, and G2/M phases (*n* = 4). All data were analyzed using independent-sample *t* tests or 2-way ANOVA and are presented as means ± SEMs. *P < 0.05, **P < 0.01, ***P < 0.001.

To investigate the impact of LRP1-ICD on HBSMC function, we constructed a recombinant plasmid overexpressing LRP1-ICD and transfected it into cells. Western blotting was used to verify the transfection efficiency. Surprisingly, we found that overexpression of LRP1-ICD significantly inhibited protein expression of the LRP1 β chain in HBSMCs compared with the group transfected with the empty vector (Figure 3, A and B). To verify that elevated LRP1-ICD may suppress full-length LRP1, through a negative feedback mechanism, we further assessed its impact on the levels of the LRP1 α chain. The results showed that, compared with the empty vector transfection group, LRP1-ICD overexpression also significantly reduced the protein levels of the LRP1 α chain in HBSMCs, supporting our hypothesis (Figure 3, C and D). Later functional assays indicated that LRP1-ICD overexpression also significantly inhibited HBSMC cell proliferation and cell cycle progression, consistent with the results observed with *LRP1* knockdown (Figure 3, E–I).

### Effects of LRP1-ICD overexpression on HBSMC function are related to the full-length LRP1.

**Figure 4:**
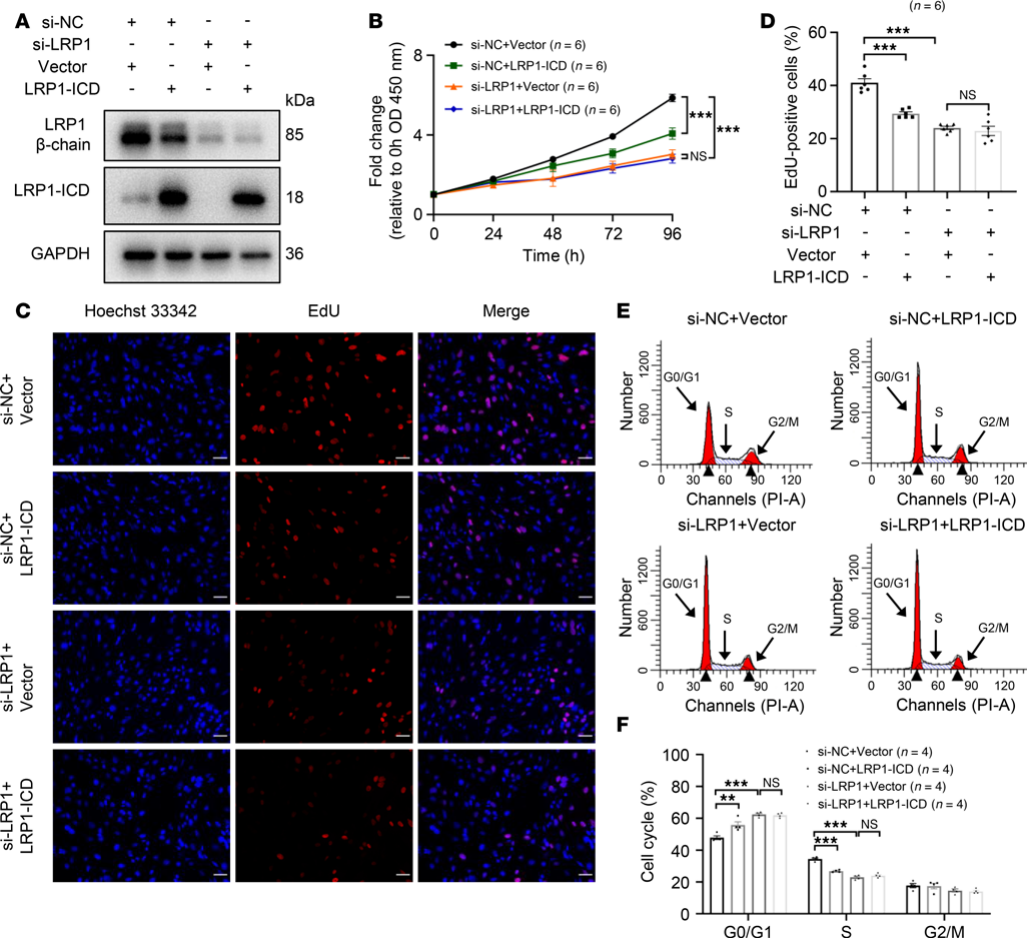
Effects of LRP1-ICD overexpression on HBSMC function are related to the full-length LRP1. (**A**) HBSMCs were transfected with *LRP1*-targeting siRNA (si-LRP1) or negative control siRNA (si-NC), along with recombinant plasmids overexpressing LRP1-ICD (LRP1-ICD) or empty vector (Vector). Representative immunoblot images showing LRP1 protein levels. (**B**) Statistical curves from the CCK-8 assay showing the cell proliferation rate (*n* = 6). (**C** and **D**) Representative fluorescence images (**C**) and summary data (**D**) from the EdU incorporation assay showing the proportion of proliferating cells (*n* = 6). Scale bar = 50 μm. (**E** and **F**) Representative histograms (**E**) and summary data (**F**) showing the proportion of cells in G0/G1, S, and G2/M phases (*n* = 4). All data were analyzed using 1- or 2-way ANOVA and are presented as means ± SEMs. **P < 0.01, ***P < 0.001.

Given the reduced α chain and β chain levels following ICD overexpression, we speculated that the full-length LRP1, rather than LRP1-ICD, is the key regulator of smooth muscle cell proliferation. To examine this hypothesis, we simultaneously knocked down *LRP1* while overexpressing LRP1-ICD to eliminate the interference caused by the effect of ICD on LRP1 protein levels, enabling us to independently observe the impact of LRP1-ICD on cell proliferation. Transfection efficiency was verified by Western blotting (Figure 4A). The results showed that *LRP1* knockdown exerted a more pronounced inhibitory effect than LRP1-ICD overexpression on cell proliferation and cell cycle progression (Figure 4, B–F). Furthermore, in the presence of *LRP1* knockdown, overexpression of LRP1-ICD did not lead to additional inhibition of cell proliferation or further alteration in the cell cycle phase distribution (Figure 4, B–F). These results demonstrate that the effects of LRP1-ICD overexpression on HBSMC function are associated with a reduction in full-length LRP1 protein levels.

### FGF2 is a downstream molecule potentially regulated by LRP1.

**Figure 5:**
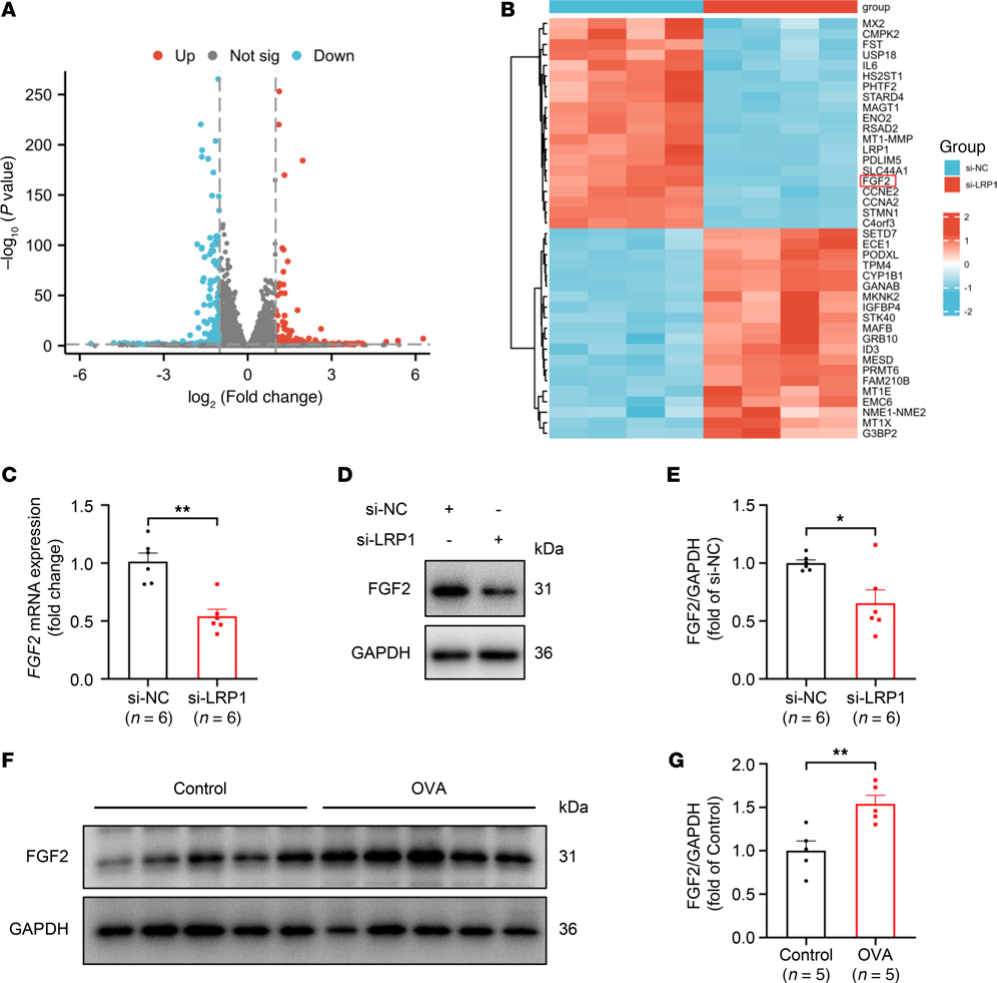
FGF2 is a downstream molecule potentially regulated by LRP1. (**A**) HBSMCs were transfected with either *LRP1*-targeting siRNA (si-LRP1) or negative control siRNA (si-NC) for 48 hours. Gene expression was assessed using high-throughput RNA sequencing. Volcano plot showing differentially expressed genes (DEGs) between the 2 groups, with selection thresholds of the absolute values of the log_2_ fold-change ≥ 1 and *P* < 0.05. Red dots represent upregulated DEGs, blue dots represent downregulated DEGs, and gray dots represent genes with no significant change (*n* = 4). (**B**) Heatmap showing the top 20 upregulated and top 20 downregulated DEGs ranked by fold-change (*n* = 4). (**C**) Summary data showing the relative expression levels of *FGF2* mRNA in indicated cells. *GAPDH* was used as an internal reference (*n* = 6). (**D** and **E**) Representative immunoblot images (**D**) and summary data (**E**) showing FGF2 protein levels in indicated cells (*n* = 6). (**F** and **G**) Representative immunoblot images (**F**) and summary data (**G**) showing FGF2 protein levels in tracheal tissues of control mice (Control) and mice with OVA-induced chronic asthma (OVA). *n* = 5 mice per group. All data were analyzed using independent-sample *t* tests and are presented as means ± SEMs. *P < 0.05, **P < 0.01.

To elucidate the specific downstream mechanisms by which LRP1 regulates cell proliferation, we utilized high-throughput RNA sequencing (RNA-Seq) to compare gene expression profiles in the *LRP1* siRNA–transfected group with those in the NC siRNA–transfected group. The results identified 494 differentially expressed genes (DEGs), including 279 downregulated and 215 upregulated genes, with selection thresholds of the absolute values of the log_2_ fold-change ≥ 1 and *P* < 0.05 (Figure 5A). After removing genes without a gene name and those with transcripts per million (TPM) < 10 in each sample, we focused on the top 20 upregulated genes and the top 20 downregulated genes based on fold-change ranking (Figure 5B). Among these 40 genes, we ultimately selected FGF2 as a candidate downstream target molecule of LRP1, because it is enriched in multiple proliferation-related signaling pathways and previous studies have shown its key role in airway remodeling in asthma (26–28). The results of qRT-PCR and Western blotting analyses conducted for validation showed that *FGF2* mRNA and FGF2 protein levels in HBSMCs transfected with *LRP1*-specific siRNA were significantly lower than those in the NC siRNA–transfected HBSMCs, consistent with our RNA-Seq results (Figure 5, C–E). LRP1-ICD overexpression also significantly reduced FGF2 protein levels compared with the empty vector transfection group (Supplemental Figure 3, A and B). However, on the basis of *LRP1* knockdown, overexpression of the ICD fragment did not exert additional inhibitory effects on FGF2 protein levels (Supplemental Figure 3, C and D). Moreover, we observed that FGF2 protein expression was significantly elevated in the tracheal tissues of mice with OVA-induced chronic asthma compared with control mice (Figure 5, F and G). These results suggest that FGF2 is a downstream molecule potentially regulated by LRP1.

### LRP1 affects HBSMC function by regulating FGF2 expression.

**Figure 6:**
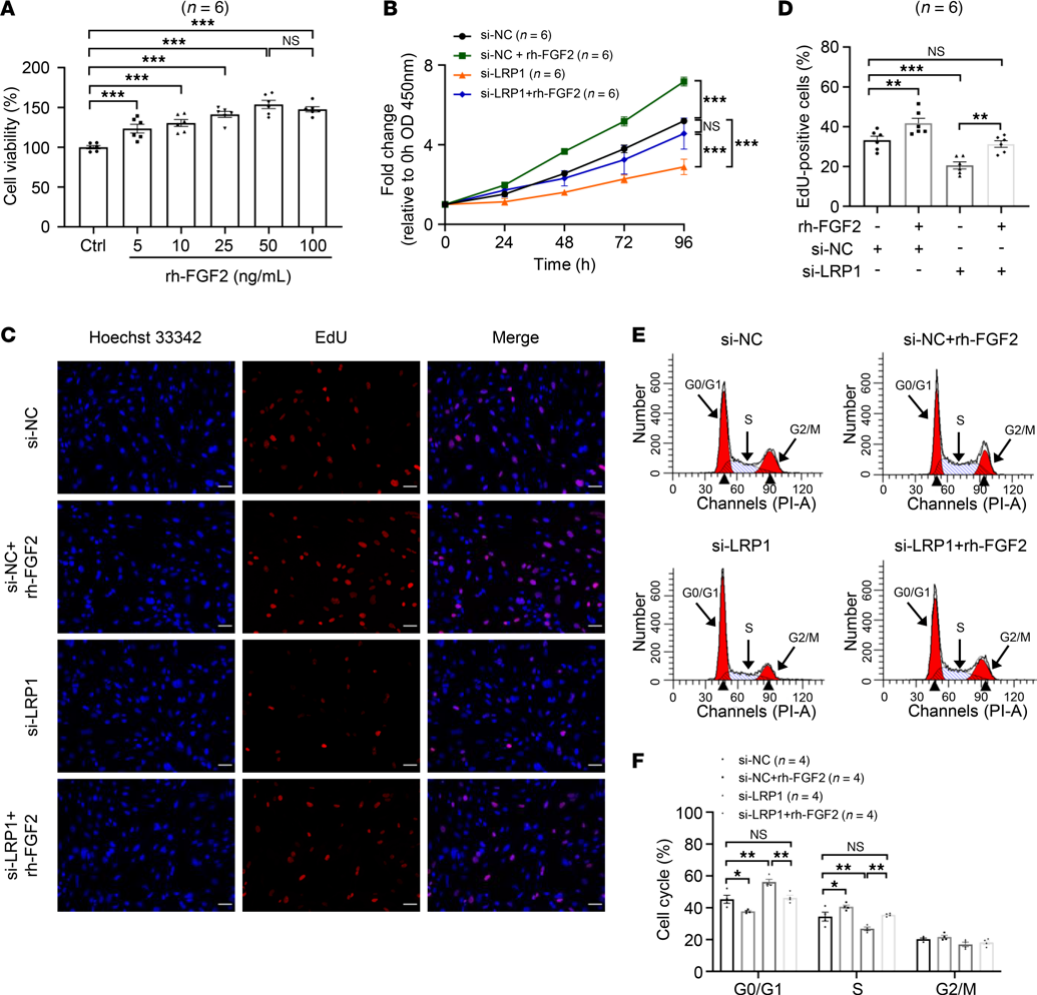
LRP1 affects HBSMC function by regulating FGF2 expression. (**A**) HBSMCs were treated with various concentrations of recombinant human FGF2 (rh-FGF2) (0, 5, 10, 25, 50, and 100 ng/mL) for 48 hours. Summary data showing cell viability (*n* = 6). (**B**) HBSMCs were transfected with *LRP1*-targeting siRNA (si-LRP1) or negative control siRNA (si-NC), with or without rh-FGF2 (50 ng/mL) treatment. Statistical curves from the CCK-8 assay showing the cell proliferation rate (*n* = 6). (**C** and **D**) Representative fluorescence images (**C**) and summary data (**D**) from the EdU incorporation assay showing the proportion of proliferating cells (*n* = 6). Scale bar = 50 μm. (**E** and **F**) Representative histograms (**E**) and summary data (**F**) showing the proportion of cells in G0/G1, S, and G2/M phases (*n* = 4). All data were analyzed using 1- or 2-way ANOVA and are presented as means ± SEMs. *P < 0.05, **P < 0.01, ***P < 0.001.

To verify whether FGF2 is involved in LRP1-mediated changes in HBSMC functions, we first supplemented the cells’ culture medium with varying concentrations of recombinant human FGF2 (rh-FGF2) for 48 hours. Cell viability was then assessed using the CCK-8 assay. The results demonstrated that rh-FGF2 promoted cell proliferation in a concentration-dependent manner, with the strongest proliferative effect observed at a concentration of 50 ng/mL. Thus, this concentration was selected for subsequent experiments (Figure 6A). We then designed a rescue experiment in which HBSMCs transfected with *LRP1* siRNA were treated with rh-FGF2. The results of a CCK-8 assay (Figure 6B) and an EdU incorporation assay (Figure 6, C and D) indicated that rh-FGF2 restored the inhibitory effect of *LRP1* siRNA on cell proliferation. Cell cycle analysis revealed that rh-FGF2 treatment significantly reduced the proportion of cells in the G0/G1 phase while significantly increasing the proportion in the S phase (Figure 6, E and F). Moreover, concurrent *LRP1* knockdown and rh-FGF2 treatment resulted in substantial recovery of G0/G1 and S phase cell proportions, reaching levels comparable to those observed in the NC siRNA–transfected group (Figure 6, E and F). These results demonstrate that LRP1 modulates the expression of FGF2 to thereby influence the function of HBSMCs.

### LRP1 knockdown inhibits the MAPK signaling pathway by suppressing FGF2 expression.

**Figure 7:**
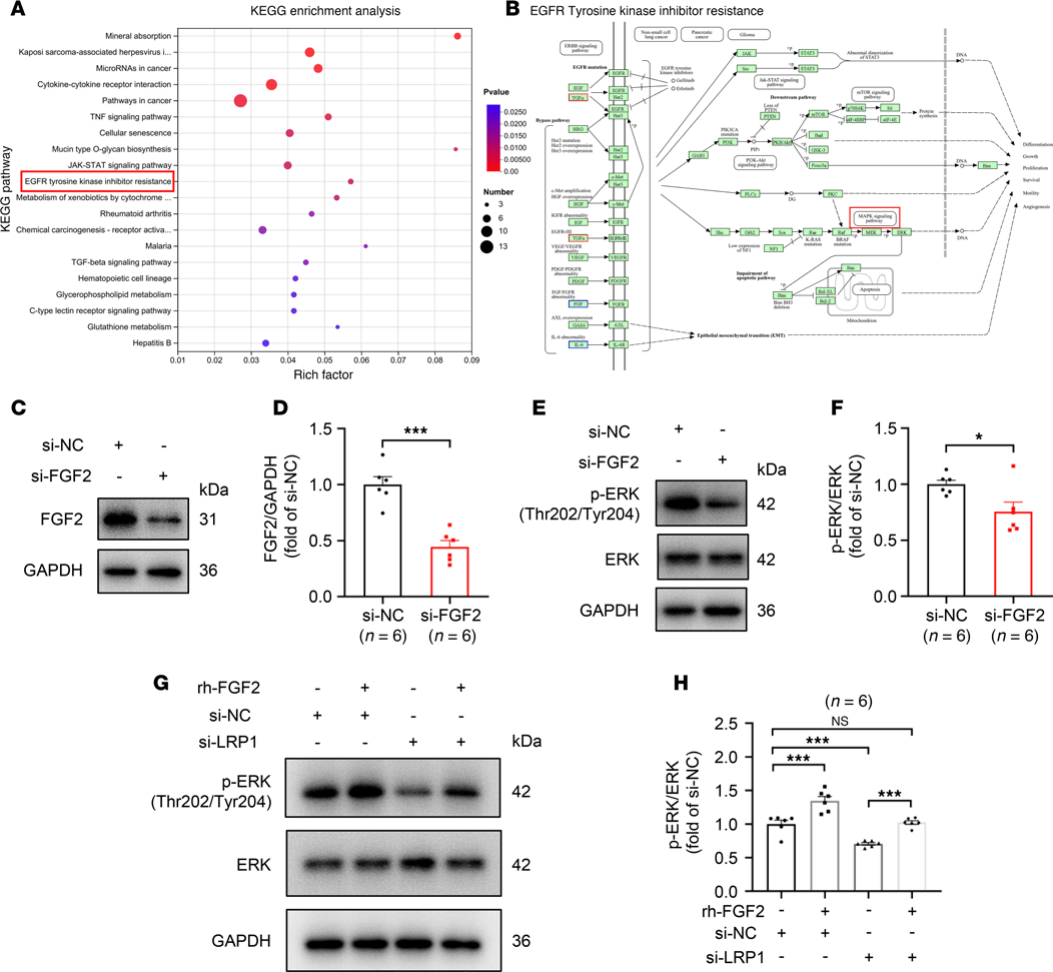
*LRP1* knockdown inhibits the MAPK signaling pathway by suppressing FGF2 expression. (**A**) HBSMCs were transfected with *LRP1*-targeting siRNA (si-LRP1) or negative control siRNA (si-NC) for 48 hours. Gene expression was assessed using high-throughput RNA sequencing (RNA-Seq). Bubble chart showing the top 20 Kyoto Encyclopedia of Genes and Genomes (KEGG) pathways (*P* < 0.05) enriched with DEGs (*n* = 4). (**B**) Pathway map showing the enrichment of DEGs from RNA-Seq in the EGFR tyrosine kinase inhibitor resistance pathway. (**C**–**F**) HBSMCs were transfected with *FGF2*-targeting siRNA (si-FGF2) or negative control siRNA (si-NC). Representative immunoblot images and summary data showing the protein levels of FGF2 (**C** and **D**), phosphorylated (p-) ERK (Thr202/Tyr204), and total ERK (**E** and **F**) in indicated cells (*n* = 6). (**G** and **H**) HBSMCs were transfected with *LRP1*-targeting siRNA (si-LRP1) or negative control siRNA (si-NC), with or without recombinant human FGF2 (rh-FGF2) (50 ng/mL) treatment. Representative immunoblot images (**G**) and summary data (**H**) showing protein levels of p-ERK (Thr202/Tyr204) and total ERK in indicated cells (*n* = 6). All data were analyzed using independent-sample *t* tests or 1-way ANOVA and are presented as means ± SEMs. *P < 0.05, ***P < 0.001.

To further elucidate the mechanisms by which LRP1 affects the functionality of HBSMCs, we conducted an in-depth analysis of the sequencing data. Figure 7A displays the top 20 Kyoto Encyclopedia of Genes and Genomes (KEGG) pathways enriched with DEGs from the RNA-Seq results that met the criterion of *P* < 0.05. Among these 20 KEGG pathways, FGF2 was enriched in 4 pathways: Kaposi sarcoma-associated herpesvirus infection (*P* = 0.00090), pathways in cancer (*P* = 0.00356), EGFR tyrosine kinase inhibitor resistance (*P* = 0.00854), and chemical carcinogenesis-receptor activation (*P* = 0.01816). Among these pathways, the EGFR tyrosine kinase inhibitor resistance pathway is closely related to cell proliferation; thus, we focused on this pathway. The pathway map suggested that FGF2 may regulate various cellular functions, such as differentiation, proliferation, motility, and angiogenesis, through the JAK/STAT, mTOR, PI3K/AKT, or MAPK signaling pathways (Figure 7B). Given that the downstream target signaling pathways we sought must be regulated by both LRP1 and FGF2, we chose the PI3K/AKT and MAPK signaling pathways for preliminary validation from the 4 identified pathways, based on previous studies (16, 29–35). The results indicated that, compared with HBSMCs transfected with NC siRNA, the phosphorylation of ERK (Thr202/Tyr204) was significantly inhibited in HBSMCs transfected with *LRP1* siRNA, while no significant change was observed in the phosphorylation of AKT (Ser473) (Supplemental Figure 4, A–D). Therefore, we conducted further validation of the MAPK signaling pathway.

We transfected HBSMCs with *FGF2*-targeting siRNA and verified the knockdown efficiency using Western blotting (Figure 7, C and D). The results showed that *FGF2*-specific siRNA transfection significantly inhibited the phosphorylation of ERK in HBSMCs (Figure 7, E and F). Subsequently, we designed a rescue experiment wherein HBSMCs transfected with *LRP1* siRNA were concurrently treated with rh-FGF2. We found that rh-FGF2 treatment reversed the inhibitory effect of *LRP1* knockdown on the MAPK signaling pathway (Figure 7, G and H). These results demonstrate that LRP1 influences the MAPK signaling pathway by regulating the expression of FGF2.

### LRP1-ICD overexpression promotes protein degradation of the LRP1 via the lysosomal pathway.

**Figure 8:**
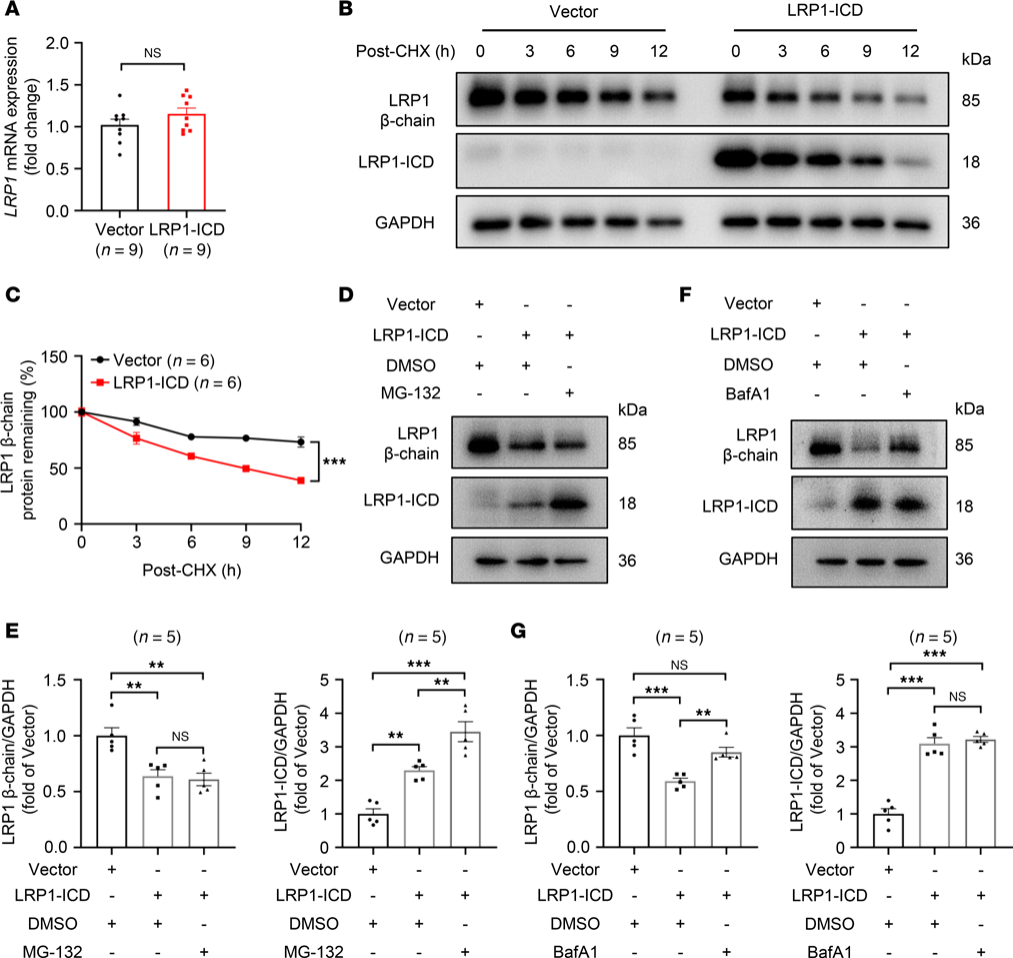
LRP1-ICD overexpression promotes protein degradation of the LRP1 via the lysosomal pathway. (**A**) HBSMCs were transfected with recombinant plasmids overexpressing LRP1-ICD (LRP1-ICD) or empty vector (Vector). Summary data showing the relative expression levels of *LRP1* mRNA in indicated cells. *GAPDH* was used as an internal reference (*n* = 9). (**B** and **C**) After HBSMCs were transfected with the target plasmid (LRP1-ICD) or empty vector (Vector) for 48 hours, the cells were treated with cycloheximide (CHX) (100 μg/mL) and harvested at different time points after treatment (0, 3, 6, 9, and 12 hours). Representative immunoblot images (**B**) and summary data (**C**) showing LRP1 protein levels (*n* = 6). (**D** and **E**) After HBSMCs were transfected with the target plasmid (LRP1-ICD) or empty vector (Vector) for 48 hours, the cells were treated with DMSO or MG-132 (10 μM) for 12 hours. Representative immunoblot images (**D**) and summary data (**E**) showing LRP1 protein levels (*n* = 5). (**F** and **G**) After HBSMCs were transfected with the target plasmid (LRP1-ICD) or empty vector (Vector) for 48 hours, the cells were treated with DMSO or bafilomycin A1 (BafA1, 200 nM) for 12 hours. Representative immunoblot images (**F**) and summary data (**G**) showing LRP1 protein levels (*n* = 5). All data were analyzed using independent-sample *t* tests or 2-way ANOVA and are presented as means ± SEMs. **P < 0.01, ***P < 0.001.

To elucidate the mechanism underlying the reduction of full-length LRP1 levels following LRP1-ICD overexpression, we transfected plasmids overexpressing LRP1-ICD into HBSMCs and then assessed the relative expression levels of *LRP1* mRNA using qRT-PCR. The results indicated that, compared with the empty vector transfection, LRP1-ICD overexpression did not suppress *LRP1* mRNA levels in HBSMCs; instead, there was a slight upward trend in expression, though the difference was not statistically significant (Figure 8A). Subsequently, we performed a cycloheximide (CHX) assay to determine whether LRP1-ICD overexpression reduced LRP1 protein levels by promoting its degradation. The results indicated that, with prolonged CHX treatment, the rate of degradation of the LRP1 in HBSMCs overexpressing LRP1-ICD was significantly accelerated compared with that of the group with the empty vector transfection (Figure 8, B and C). The above results suggest that the elevated LRP1-ICD inhibits LRP1 expression by promoting its protein degradation, rather than suppressing its transcription.

Degradation of proteins generally occurs through the ubiquitin-proteasome pathway or the lysosomal degradation pathway. To further investigate the underlying mechanism of LRP1 degradation, we treated HBSMCs with a proteasome inhibitor (MG-132) and a lysosomal inhibitor (bafilomycin A1). The results showed that the LRP1-ICD protein level in MG-132–treated HBSMCs was significantly higher than in the DMSO-treated group (Figure 8, D–G). However, the increase in LRP1 degradation mediated by LRP1-ICD overexpression was blocked by bafilomycin A1 pretreatment but not by MG-132 (Figure 8, D–G). These results suggest that elevated LRP1-ICD promotes the protein degradation of LRP1 via the lysosomal pathway.

### Upregulation of MT1-MMP expression in tracheal tissues of mice with OVA-induced chronic asthma.

**Figure 9:**
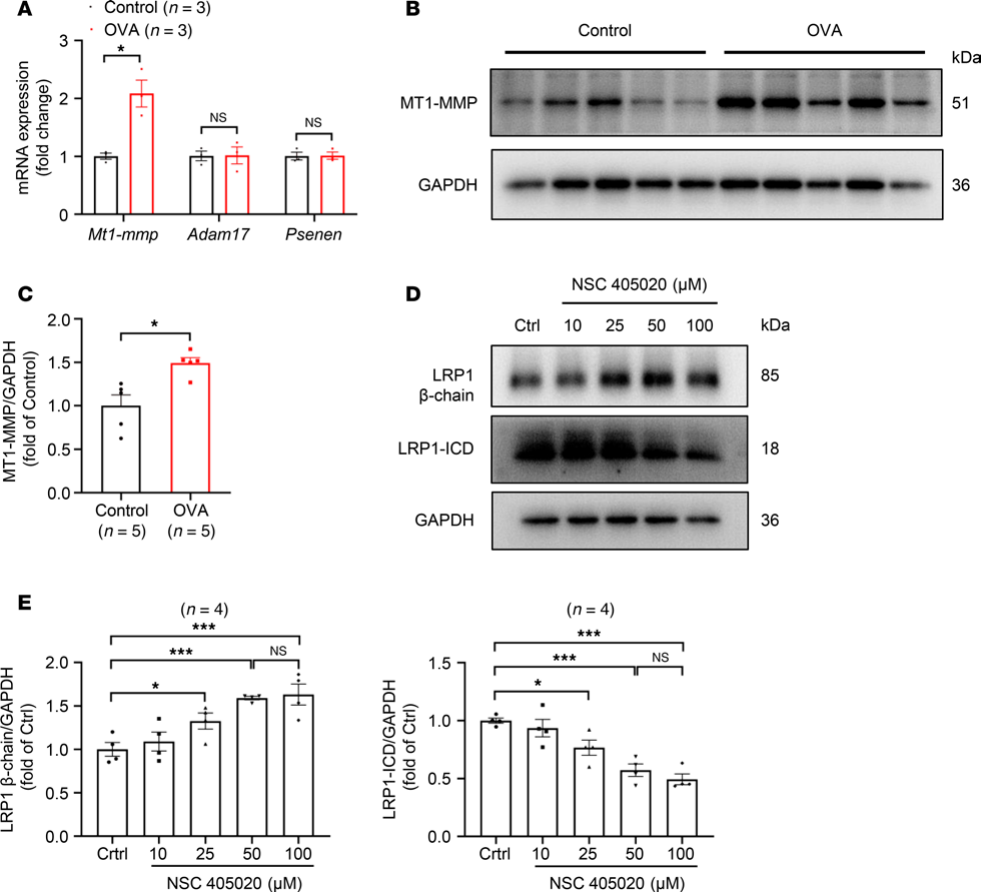
Upregulation of MT1-MMP expression in tracheal tissues of mice with OVA-induced chronic asthma. (**A**) Summary data showing the relative expression levels of *Mt1-mmp*, *Adam17*, and *Psenen* mRNA in tracheal tissues of control mice (Control) and mice with OVA-induced chronic asthma (OVA). *Gapdh* was used as an internal reference. *n* = 3 mice per group. (**B** and **C**) Representative immunoblot images (**B**) and summary data (**C**) showing MT1-MMP protein levels in tracheal tissues of both groups of mice. *n* = 5 mice per group. (**D** and **E**) HBSMCs were treated with various concentrations of NSC 405020 (0, 10, 25, 50, and 100 μM) for 24 hours. Representative immunoblot images (**D**) and summary data (**E**) showing LRP1 protein levels (*n* = 4). All data were analyzed using independent-sample *t* tests and are presented as means ± SEMs. *P < 0.05, ***P < 0.001.

Since LRP1-ICD is a small fragment produced by proteolysis of the LRP1, its levels should theoretically change in the same direction and proportion as the β chain. However, we observed that in the tracheal tissues of mice modeling chronic asthma, the elevation in ICD levels was significantly greater than that of the β chain (Figure 1, G and H). This discrepancy may be attributed to the activation of LRP1 proteolytic processing during asthma, which would lead to the generation of more ICD fragments. Accordingly, we examined the expression levels of 3 key enzymes involved in the production of ICD: MT1-MMP, ADAM17, and γ-secretase. The results from our qRT-PCR analyses showed that the expression level of *Mt1-mmp* mRNA was significantly higher in tracheal tissues of mice with OVA-induced asthma compared with control mice (Figure 9A). By contrast, *Adam17* and *Psenen* (catalytic subunit of γ-secretase) mRNA levels showed no significant difference between the 2 groups (Figure 9A). Western blotting results verified that the protein expression of MT1-MMP in the tracheal tissues of mice with OVA-induced asthma was significantly higher than that in control mice (Figure 9, B and C). To verify the role of MT1-MMP in the proteolytic processing of LRP1, we treated HBSMCs with NSC 405020. NSC 405020 is a specific inhibitor of MT1-MMP, which directly interacts with the hemopexin domain of MT1-MMP (35, 36). We found that NSC 405020 inhibited LRP1 proteolysis in a concentration-dependent manner, as evidenced by the increase in LRP1 β chain protein levels and the decrease in LRP1-ICD protein levels with increasing inhibitor concentrations (Figure 9, D and E). These results demonstrate that the transcriptional activation of *Mt1-mmp* may be responsible for the abnormal increase of LRP1-ICD in the tracheal tissues of mice with asthma.

### Lrp1 knockdown attenuates ASM proliferation in mice with OVA-induced chronic asthma.

**Figure 10:**
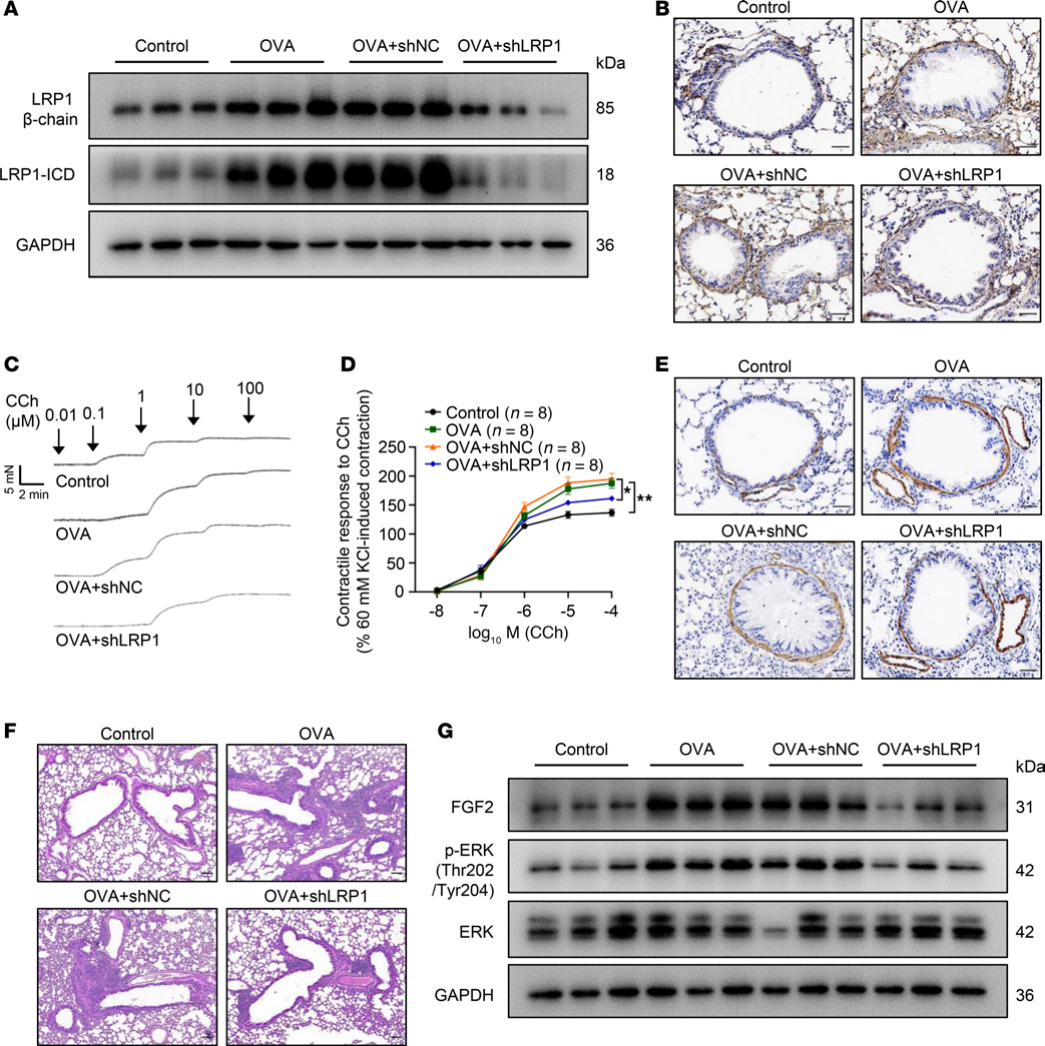
*Lrp1* knockdown attenuates ASM proliferation in mice with OVA-induced chronic asthma. (**A**) Mice were assigned to 1 of 4 groups: control, OVA-induced chronic asthma (OVA), OVA treatment concurrent with intratracheal instillation of lentivirus containing negative control shRNA (OVA+shNC), and OVA treatment concurrent with intratracheal instillation of lentivirus containing *Lrp1*-targeting shRNA (OVA+shLRP1). Representative immunoblot images showing LRP1 protein levels in tracheal tissues of the 4 groups. (**B**) Representative immunohistochemistry images showing LRP1 protein levels in ASM of the 4 groups of mice. Scale bar: 50 μm. (**C** and **D**) Representative traces (**C**) and statistical curves (**D**) showing contractile responses of tracheal rings to cumulative concentrations of carbachol (CCh) (10^–8^ to 10^–4^ mol/L) in the 4 groups of mice. *n* = 8 tracheal rings from 4 mice per group. (**E**) Representative immunohistochemistry images showing α-SMA protein levels in lung tissues derived from the 4 groups of mice. Scale bar: 50 μm. (**F**) Representative images of H&E staining of lung tissues from the 4 groups of mice. Scale bar: 100 μm. (**G**) Representative immunoblot images showing protein levels of FGF2, phosphorylated (p-) ERK (Thr202/Tyr204), and total ERK in tracheal tissues of the 4 groups of mice. All data were analyzed by 1- or 2-way ANOVA and are presented as means ± SEMs. *P < 0.05, **P < 0.01.

To verify the critical role of LRP1 in ASM proliferation in chronic asthma in vivo, we administered intratracheal instillations of lentivirus containing *Lrp1* shRNA or NC shRNA to OVA-challenged mice. Western blotting results suggested that, compared with the NC shRNA–treated group, *Lrp1* shRNA significantly reduced the protein levels of the β chain and ICD in the tracheal tissues of OVA-treated mice (Figure 10A and Supplemental Figure 5A). Immunohistochemistry results similarly indicated that the expression level of LRP1 in ASM cells of mice with OVA-induced asthma treated with *Lrp1* shRNA was significantly lower than that in the mice treated with NC shRNA (Figure 10B and Supplemental Figure 5B). Ex vivo tracheal tension measurement experiments indicated that the tracheal contractile response to carbachol was significantly reduced after application of *Lrp1* shRNA compared with NC shRNA in mice with OVA-induced asthma (Figure 10, C and D). Alpha–smooth muscle actin (α-SMA) is a protein marker of smooth muscle cells, and the area of α-SMA^+^ staining in the bronchial wall can reflect the extent of ASM proliferation. The α-SMA–stained area in the bronchial wall was significantly increased in mice with OVA-induced asthma compared with control mice and was significantly decreased in OVA-challenged mice after application of *Lrp1* shRNA compared with NC shRNA (Figure 10E and Supplemental Figure 5C). Interestingly, we found that *Lrp1* shRNA also significantly alleviated OVA-induced peribronchial inflammatory infiltration compared with NC shRNA–treated mice (Figure 10F and Supplemental Figure 5D). Furthermore, we found that *Lrp1* knockdown significantly reduced the levels of FGF2 and phosphorylated ERK compared with NC shRNA treatment in the tracheal tissues of mice with OVA-induced asthma, consistent with our in vitro findings (Figure 10G and Supplemental Figure 5E). These results demonstrate that suppression of *Lrp1* alleviates ASM proliferation, airway hyperresponsiveness, and airway inflammation in mice with OVA-induced chronic asthma.

### LRP1 regulates the expression of MT1-MMP.

The aforementioned findings suggest that MT1-MMP is involved in the proteolytic process of LRP1. Interestingly, from the RNA-Seq results following *LRP1* knockdown, we found that LRP1 could also regulate the expression of MT1-MMP (Figure 5B). The results of qRT-PCR and Western blotting analyses conducted for validation showed that *MT1-MMP* mRNA and MT1-MMP protein levels were significantly lower in HBSMCs transfected with *LRP1*-specific siRNA compared with NC siRNA–transfected HBSMCs, consistent with the RNA-Seq results (Supplemental Figure 6, A–C). LRP1-ICD overexpression also significantly reduced MT1-MMP protein levels compared with the empty vector transfection group (Supplemental Figure 6, D and E). However, the inhibitory effect of *LRP1* knockdown was more pronounced. Moreover, on the basis of *LRP1* knockdown, overexpression of the ICD fragment did not further reduce MT1-MMP protein levels (Supplemental Figure 6, F and G). The in vivo results similarly showed that, compared with the NC shRNA–treated group, *Lrp1* shRNA significantly decreased MT1-MMP protein levels in the tracheal tissues of OVA-treated mice (Supplemental Figure 6, H and I). These results demonstrate the regulatory effect of LRP1 on the expression of MT1-MMP and suggest that the transcriptional activation of *Mt1-mmp* in tracheal tissues of mice with OVA-induced chronic asthma may be mediated by increased LRP1.

## Discussion

Asthma is a heterogeneous clinical syndrome that can affect individuals of all age groups, with a persistently high prevalence worldwide (1, 2). ASM proliferation is a key feature of airway remodeling in asthma. By generating greater force, ASM promotes airway hyperresponsiveness, leading to irreversible lung function changes in patients with asthma (9). Despite considerable advances in elucidating the inflammatory characteristics of asthma, the cellular and molecular mechanisms involved in airway remodeling remain poorly understood. The main findings of this study were as follows. (a) We identified that LRP1 was significantly increased in ASM cells of mice with OVA-induced chronic asthma, with LRP1-ICD protein levels rising to a much greater extent than those of the LRP1 β chain. (b) Knockdown of *LRP1* inhibited ERK phosphorylation by downregulating FGF2 expression, thereby arresting the cell cycle progression and inhibiting the proliferation of HBSMCs. (c) Overexpression of LRP1-ICD also significantly inhibited the proliferation of HBSMCs. However, concurrent with *LRP1* knockdown, LRP1-ICD overexpression did not further arrest the cell cycle progression or inhibit the proliferation of HBSMCs. (d) LRP1-ICD overexpression reduced protein levels of LRP1 by promoting its degradation rather than by suppressing the transcription of the *LRP1* gene. (e) *Mt1-mmp* mRNA and MT1-MMP protein levels were significantly upregulated in tracheal tissues of mice with OVA-induced asthma. (f) Inhibition of LRP1 significantly alleviated peribronchial inflammation, airway hyperreactivity, and ASM proliferation in mice with OVA-induced asthma, as well as suppressed OVA-induced increases in FGF2 and phosphorylated ERK expression. (g) Knockdown of *LRP1* significantly inhibited the expression of MT1-MMP both in vitro and in vivo. Overall, these key findings suggest that LRP1 may promote ASM proliferation in mice with OVA-induced chronic asthma by activating the FGF2/ERK signaling pathway. Elevated LRP1-ICD may negatively regulate LRP1, thus preventing further worsening of asthma because of excessive upregulation of LRP1. Hence, LRP1 holds promise as a therapeutic target for ASM remodeling in asthma.

LRP1 is a widely expressed endocytosis/signaling cell surface receptor that plays a critical role in various physiological and pathological processes (37). In the current study, we observed that LRP1 was significantly increased in the tracheal tissues of mice with OVA-induced asthma compared with control mice, as evidenced by both *Lrp1* mRNA levels (Figure 1F) and LRP1 β chain protein levels (Figure 1H) being increased, while its expression in the lung tissue of both groups showed no significant difference (Supplemental Figure 1B). Because we meticulously removed the surrounding connective tissue and epithelial layers during tracheal tissue sampling, the primary cellular components in the tracheal tissue were ASM cells. However, in addition to smooth muscle cells, the lung tissue contained a substantial number of pulmonary epithelial cells and structural cells making up the vascular walls. Therefore, we hypothesized that ASM cells may be the primary cells in which LRP1 exerts its role in the pathogenesis of asthma. Immunohistochemical results of lung tissues further verified the increased expression of LRP1 in ASM cells of mice with OVA-induced asthma.

Previous studies have shown that LRP1 is involved in regulating the proliferation of various cell types (17, 18, 31, 38, 39), but its role in ASM cell proliferation has not yet been reported. In the present study, we found that silencing *LRP1* using specific siRNA significantly inhibited the proliferation of HBSMCs. In vivo experiments also verified that suppression of *Lrp1* greatly reduced OVA-induced ASM proliferation. This result is consistent with previous reports regarding the role of LRP1 in the proliferation of other cell types (17, 18, 31). However, there are also studies that contradict our results, particularly in vascular research. For example, Au et al. found that primary descending aortic smooth muscle cells lacking *LRP1* exhibit a higher proliferation rate than normal cells (38). Similarly, Boucher et al. found that inactivation of *Lrp1* in mouse vascular smooth muscle leads to smooth muscle proliferation, suggesting that LRP1 plays a crucial role in maintaining vascular wall integrity and preventing atherosclerosis by controlling PDGF activation (39). The differences in these results indicate that the function of LRP1 is highly tissue and cell specific. Therefore, further research is needed to explore its roles in various cell types. To our knowledge, our study is the first to identify the impact of LRP1 on the proliferation of ASM cells, which provides a potential target for exploring the mechanisms underlying various respiratory diseases that could involve airway remodeling, including asthma and chronic obstructive pulmonary disease.

To further elucidate the mechanism by which LRP1 regulates ASM cell proliferation, we utilized RNA-Seq to detect changes in downstream gene expression following *LRP1* knockdown in HBSMCs. Our results indicated that the reduction in LRP1 significantly decreased FGF2 expression, both in vivo (Supplemental Figure 5E) and in vitro (Figure 5E). FGF2 is a member of the largest growth factor family, the FGF protein family (26). Currently, FGF2 has been found to promote the growth of various cell types, including ASM cells (26). Furthermore, previous studies have demonstrated that, aside from its mitogenic effects when administered alone, FGF2 pretreatment enhances the proliferative response of human ASM cells to various asthma mediators (27, 28). Multiple studies have reported increased expression of FGF2 in patients with asthma and animal models of asthma (32, 40, 41). In the present study, we similarly found that FGF2 expression was significantly higher in airway tissues of mice with OVA-induced asthma compared with control mice (*P* = 0.007) (Figure 5G). Furthermore, our study suggested a regulatory relationship between LRP1 and FGF2, which may offer new avenues for the treatment of diseases that require targeting FGF2. Several prior studies have reported the regulatory effects of LRP1 and FGF2 on the MAPK signaling pathway (16, 31–34). Here, we also observed that transfection of HBSMCs with *LRP1* siRNA or *FGF2* siRNA significantly reduced the levels of phosphorylated ERK (Figure 10). Importantly, however, we further demonstrated through rescue experiments that *LRP1* knockdown inhibited ERK phosphorylation by reducing FGF2 expression. In vivo experiments revealed that the levels of phosphorylated ERK were significantly elevated in the airway tissues of mice with OVA-induced asthma (Figure 10), consistent with multiple previous reports (42–44). Notably, we also found that interference with *Lrp1* inhibited the OVA-induced increase in phosphorylated ERK levels in airway tissues. These findings provide a potential molecular mechanism for elucidating how LRP1 regulates the proliferation of ASM cells.

Our results showed that the expression of full-length LRP1 was significantly elevated in the tracheal tissues of mice with OVA-induced asthma as evidenced by both *Lrp1* mRNA levels and LRP1 β chain protein levels being increased. Targeted inhibition of full-length LRP1 using a specific shRNA in OVA-treated mice effectively alleviated ASM remodeling, suggesting that the increase in full-length LRP1 exacerbates asthma. Meanwhile, our study found that the increased LRP1 in the ASM of mice with OVA-induced asthma may activate the transcription of *Mt1-mmp*, thereby promoting the proteolytic process of LRP1 and generating more LRP1-ICD. This explains why we observed that the increase in LRP1-ICD protein levels was significantly greater than that of the LRP1 β chain in the tracheal tissues of mice with OVA-induced asthma. LRP1-ICD is a product of stepwise proteolysis of LRP1, and its function has often been overlooked in previous studies on LRP1. In the present study, we found that elevated LRP1-ICD may reduce the levels of full-length LRP1 by promoting its degradation via the lysosomal pathway. In light of this finding, we hypothesize that the abnormal elevation of LRP1-ICD in the mouse asthma model may represent a protective mechanism, helping to prevent further exacerbation of asthma caused by excessive increases in full-length LRP1. However, the specific mechanism by which LRP1-ICD promotes the degradation of the LRP1 remains unclear. Previous studies have suggested that ICD released from the plasma membrane can translocate to the nucleus to regulate the transcription of downstream genes (24). Therefore, investigating whether LRP1-ICD translocates to the nucleus and regulates the transcription of genes associated with protein degradation pathways will be a key focus of our future research.

The main limitations of this study lie in the absence of validation from clinical specimens. Furthermore, the coding sequence of the *LRP1* gene is quite lengthy, making overexpression of the full-length protein challenging. Therefore, in the current study, we explored the function and mechanisms of LRP1 only by silencing the target gene, which lacks supporting evidence for reverse validation.

In summary, our results indicated that LRP1 was upregulated in ASM cells of mice with OVA-induced chronic asthma and that this upregulated LRP1 promoted ASM cell proliferation by activating the FGF2/ERK signaling pathway. The transcriptional activation of *Mt1-mmp* enhanced the production of LRP1-ICD, which may promote protein degradation of the LRP1 via the lysosomal pathway, thereby preventing further exacerbation of asthma. Our study provides a potential target for the prevention and treatment of ASM remodeling in patients with asthma.

## Methods

### Sex as a biological variable.

Our study exclusively examined female mice because of previous findings indicating that female mice exhibit a more pronounced response to allergens compared with male mice (45, 46). Given the well-recognized sex differences in asthma (47), future research should investigate whether our results extend to male mice.

### Mice.

Six-week-old female BALB/c mice, with a weight range of 16–20 g, were obtained from GemPharmatech. Mice were housed in an animal facility with an ambient temperature of 21°C to 25°C, a relative humidity of 50% to 70%, and a 12-hour light/12-hour dark cycle. Water and food were accessible to the mice without restriction. All mice were given a 1-week acclimatization period before the commencement of the experiments.

### Construction and therapeutic intervention of the mouse model of chronic asthma.

Mice were randomly assigned to the control, OVA, OVA treatment concurrent with intratracheal instillation of lentivirus containing negative control shRNA (OVA+shNC), and OVA treatment concurrent with intratracheal instillation of lentivirus containing *LRP1*-targeting shRNA (OVA+shLRP1) groups. On days 1, 8, and 15, mice in the OVA group were intraperitoneally injected with 200 μL of sensitizing solution containing 100 μg of OVA (A5503, Sigma-Aldrich) and 50 μL of alum adjuvant (77161, Thermo Fisher Scientific). From day 22, mice were exposed to 1% OVA by nebulized inhalation in a closed container for 30 minutes daily for 1 week. From day 29, mice were challenged with 1% OVA via nebulized inhalation every other day for 4 weeks until day 56 (Figure 1A). Meanwhile, mice in the OVA+shNC and OVA+shLRP1 groups received additional interventions, namely, intratracheal instillations of lentiviruses containing NC shRNA or target *Lrp1* shRNA on days 22 and 39. Each mouse was given 5.0 × 10^6^ transfection units per injection. The shRNA was delivered using the lentiviral vector pCLenti-U6-shRNA-CMV-EGFP-WPRE (Vector ID: GL404), which was synthesized by OBiO Technology. The target sequences used were 5′-CCTAAGGTTAAGTCGCCCTCG-3′ for shNC and 5′-AGGCGCCTGTGTGGTCAATAA-3′ for shLRP1. The control group mice received the same schedule of treatments with an equivalent volume of sterile phosphate-buffered saline (PBS). On day 57, mice were euthanized for the collection of BALF, tracheal tissues, and lung tissues for further analysis.

### Ex vivo tracheal tension measurement.

Ex vivo tracheal tension measurements were conducted according to the methods previously detailed (48). Briefly, following euthanasia of the mice with a carbon dioxide overdose, the trachea was isolated and submerged in oxygenated Krebs solution, which consisted of 118 mM NaCl, 4.7 mM KCl, 1.2 mM KH_2_PO_4_, 2.5 mM CaCl_2_, 1.2 mM MgSO_4_·7H_2_O, 25.2 mM NaHCO_3_, and 11.1 mM glucose, with a pH of 7.4. The epithelial layer was carefully removed, and the trachea was divided into 2 to 3 mm segments. Tracheal rings were then suspended in an organ bath with 5 mL of Krebs solution (37°C) that was continuously ventilated with 95% O_2_ and 5% CO_2_. Tracheal tension measurements were performed using a BL-420N Biological Signal Acquisition and Analysis System (Taimeng Technology). An initial tension of 5 mN was applied to the tracheal rings, which were then equilibrated for 30 minutes before the experiment began. The contractile response of the tracheal rings was then assessed using 60 mM K^+^ solution (replacing sodium chloride with potassium chloride in equivalent amounts). The second result was taken for subsequent analysis. Then, cumulative concentrations of carbachol (C0596, TCI) (from 10^–8^ M to 10^–4^ M) were added to the bath, and traces of the tension changes were recorded. Reported *n* values in these experiments represent the number of tracheal rings used for tension detection.

### BALF collection and cell counting.

After the mice were euthanized, 0.8 mL of sterile PBS was introduced into the trachea. Then, the lavage was performed 3 times to collect BALF from both lungs, with a fluid recovery rate of over 90%. The harvested BALF was centrifuged at 1,200*g* for 10 minutes at 4°C. Cell counting was performed after resuspending the pellet in 100 μL of PBS. Total cell counts were measured using a hemocytometer. Eosinophil counts were determined using the Wright-Giemsa staining method.

### Histological analysis.

Lung tissues from mice were fixed in 4% paraformaldehyde for at least 24 hours. The tissue samples were then dehydrated, paraffin-embedded, and sectioned into 4 μm–thick slices. H&E staining was used to assess peribronchial inflammatory infiltration according to the following scoring system: no inflammatory cell infiltration (score 0), a few inflammatory cells (score 1), a ring of inflammatory cells with a depth of single cell layer (score 2), a ring of inflammatory cells with a depth of 2 to 4 cells (score 3), and a ring of inflammatory cells with a depth of more than 4cells (score 4) (49). PAS staining was used to assess goblet cell hyperplasia in the bronchial mucosa according to the previously described following scoring system: no red staining (score 0), red staining involving < 25% of the epithelium (score 1), red staining involving 25%–50% of the epithelium (score 2), red staining involving 50%–75% of the epithelium (score 3), and red staining involving > 75% of the epithelium (score 4) (49). Masson’s trichrome staining (Masson staining) was employed to evaluate peribronchial collagen deposition, and relative collagen content was determined using ImageJ software (NIH). Three images were analyzed per section at 100× original magnification, and their average was recorded as the section’s final result. Each group included 5 biological replicates.

### Immunohistochemical staining.

To detect protein expression and distribution in tissues, paraffin-embedded tissue sections were processed to remove paraffin and then rehydrated. The sections sequentially underwent antigen retrieval in citrate buffer at pH 6.0, were treated with 3% hydrogen peroxide solution to block endogenous peroxidase activity, and were treated with 3% BSA to block nonspecific binding. Following this, the sections were incubated overnight at 4°C with primary antibodies specific to LRP1 (1:50; ab92544, Abcam) and α-SMA (1:500; GB111364-100, Servicebio). After 3 washes with PBS, the sections were incubated with horseradish peroxidase–conjugated secondary antibody (1:200; GB23303, Servicebio) at room temperature for 50 minutes. Diaminobenzidine was used as the chromogen, followed by counterstaining with hematoxylin. Brown staining was considered positive expression. Results were analyzed using ImageJ software. LRP1 protein levels in the ASM were expressed as the percentage of the area that was LRP1 positive in the bronchial smooth muscle layer of lung tissue sections. Immunohistochemical staining for α-SMA was used to reflect the degree of ASM proliferation. Results were reported as the ratio of peribronchial α-SMA staining area to the basement membrane length of bronchioles.

### RNA extraction and qRT-PCR.

TRIzol reagent (DP424, Tiangen) was utilized to isolate total RNA from cells and tissues, and the concentration of extracted RNA was detected by a NanoDrop One spectrophotometer (Thermo Fisher Scientific). Subsequently, a reverse transcription kit (22107, Tolobio) was used to convert 1 μg of RNA into cDNA. We performed qRT-PCR analysis of cDNA with SYBR qPCR Master Mix (22204, Tolobio) by using an ABI QuantStudio 6 Flex system. GAPDH was employed as the internal reference. Relative mRNA expression levels were determined using the 2^–ΔΔCt^ method. Details for all primers used in this study are provided in Supplemental Table 1.

### Western blotting.

Total protein was extracted from cells and tissues using RIPA lysis buffer (P0013B, Beyotime), and the protein concentration was detected using a BCA assay kit (P0012S, Beyotime). Equal amounts of protein were separated by 12% or 6% sodium dodecyl sulfate–polyacrylamide gel electrophoresis, depending on the molecular weight of the target protein, and then transferred onto PVDF membranes (ISEQ00010, MilliporeSigma). Next, the membranes were blocked with 5% skimmed milk at room temperature for 1 hour, followed by incubation with primary antibodies at 4°C overnight. The primary antibodies used included anti-LRP1 β chain (1:30,000; ab92544, Abcam), anti–LRP1-ICD (1:5,000; ab92544, Abcam), anti-LRP1 α chain (1:2,000; 30206-1-AP, Proteintech), anti-FGF2 (1:500; 11234-1-AP, Proteintech), anti–phosphorylated ERK1/2 (Thr202/Tyr204) (1:1,000; AF1015, Affinity Biosciences), anti-ERK1/2 (1:10,000; 11257-1-AP, Proteintech), anti–phosphorylated AKT (Ser473) (1:2,000; 66444-1-Ig, Proteintech), anti-AKT (1:5,000, 10176-2-AP, Proteintech), anti–MT1-MMP (1:1,000; 29111-1-AP, Proteintech), and anti-GAPDH (1:10,000; AF7021, Affinity Biosciences). On the second day, the membranes were incubated with horseradish peroxidase–conjugated secondary antibody (1:5,000; E-AB-1003 for rabbit primary antibody and E-AB-1001 for mouse primary antibody, Elabscience) at room temperature for 2 hours. Detection was performed using chemiluminescence (Abbkine, model BMU101-EN). GAPDH was used as the internal reference, and protein bands were analyzed using ImageJ software.

### Cell culture.

Immortalized HBSMCs were purchased from iCell Bioscience (iCell-0121a). Cells were cultured in DMEM (BC-M-005, Bio-Channel) supplemented with 15% fetal bovine serum and 1% penicillin/streptomycin and maintained in an incubator at 37°C with 5% CO_2_.

### RNA interference.

The siRNAs were synthesized by Sangon Biotech. The siRNA sequences used in this study are as follows: si-NC, 5′-UUCUCCGAACGUGUCACGUTT-3′ (sense strand); si-LRP1, 5′-GCGAACAAACACACUGGCUAATT-3′ (sense strand); si-FGF2, 5′-CUAUCAAAGGAGUGUGUGCUATT-3′ (sense strand). Cells were cultured in 12-well plates until 60% confluence. Then, cells were transfected with 200 nmol/L siRNA for 48 hours using 2 μL of Lipofectamine 3000 (L3000015, Thermo Fisher Scientific). Western blotting was employed to verify the transfection efficiency.

### Plasmid transfection.

Recombinant plasmids overexpressing LRP1-ICD were constructed by Sangon Biotech. The sequence was based on a study reported by Zurhove et al. and comprised nucleotides 13,746–14,102 of NM_002332.3 preceded by a Kozak sequence (24). The target gene was synthesized using whole-gene synthesis technology and inserted into the pcDNA3.1 vector. Cells were cultured in 12-well plates until 70% confluence. Then, cells were transfected with 0.3 μg of plasmid for 48 hours using 0.5 μL Lipofectamine 3000 and 0.6 μL of p3000. The empty vector (pcDNA 3.1) was used as the control. Western blotting was employed to verify the transfection efficiency.

### Cell viability assay.

Cell viability was examined with CCK-8 kits (C0005, TargetMol) as per the manufacturer’s guidelines. Transfected or untreated cells were seeded in 96-well plates, with 10,000 cells per well, in a volume of 100 μL of culture medium. Cells were treated with rh-FGF2 (AF-100-18B, PeproTech) or without drugs, depending on the requirements of each experiment. Cells were allowed to grow for different periods (0, 24, 48, 72, and 96 hours). Next, each well received 10 μL of CCK-8 reagent, and the cells were incubated for 2 hours at 37°C with 5% CO_2_. The values of absorbance at 450 nm were recorded.

### EdU incorporation assay.

The incorporation of EdU in proliferating cells was measured using an EdU detection kit (C0071S, Beyotime). Cells were seeded on poly-l-lysine–coated, round coverslips in 12-well plates. Cells were transfected and treated with or without drugs according to the requirements of each experiment. Cells were then exposed to 10 μM EdU at 37°C for 2 hours. Next, cells underwent fixation with 4% paraformaldehyde and were permeabilized with 0.3% Triton X-100. Following this, each well of cells was incubated with 200 μL of the click reaction reagent (part of the EdU detection kit) away from light for 30 minutes. Hoechst 33342 was used to stain the nuclei. Representative images were captured with an upright fluorescence microscope (DM6B, Leica). ImageJ software was used to analyze the percentage of EdU-positive cells.

### Flow cytometry for cell cycle analysis and apoptosis detection.

Cells were seeded in 6-well plates and subjected to various treatments. Cell cycle analysis was conducted with a propidium iodide (PI) Cell Cycle Detection Kit (BB4104, Bestbio). Briefly, cells collected by trypsinization were washed with PBS, resuspended in 75% ethanol, and stored at –20°C overnight. On the following day, centrifugation at 1,000*g* removed the ethanol, and we washed the cells with PBS. We then treated the cells with PI staining solution at 4°C away from light for 30 minutes. The distribution of cells across the G0/G1, S, and G2/M phases was measured by a flow cytometer (CytoFLEX, Beckman Coulter Life Sciences). Data were analyzed with ModFit software.

Apoptosis was analyzed with an Annexin V-FITC/PI double staining kit (BB4101, Bestbio). Briefly, cells collected by trypsinization were washed with PBS. Next, the cell suspension was incubated with Annexin V-FITC and PI. The proportion of apoptotic cells was measured using a flow cytometer (CytoFlex). Data were analyzed with FlowJo software.

### RNA-Seq analysis.

HBSMCs were transfected with either *LRP1*-targeting siRNA (si-LRP1) or NC siRNA (si-NC) for 48 hours, and total RNA isolation was performed with TRIzol reagent. Subsequently, mRNA was purified from total RNA by using Oligo (dT)–coated magnetic beads (Yeasen, catalog 19820ES). The mRNA was then randomly fragmented into approximately 300–base pair segments and reverse-transcribed to cDNA. The TruSeq RNA sample preparation kit (Illumina) was used for library construction. Sequencing was conducted on the Illumina NovaSeq 6000 platform. TPM was employed to quantify gene expression levels, and DEGs were analyzed with DESeq2 software. Genes were defined as DEGs if they met the criteria of the absolute values of the log_2_ fold-change ≥ 1 and *P* < 0.05. KEGG pathway enrichment analysis was carried out with the KOBAS online analysis database (http://bioinfo.org/kobas), with *P* < 0.05 considered statistically significant.

### Statistics.

Statistical analysis was performed using SPSS 26.0 software. For continuous variables, the Kolmogorov-Smirnov test was used to assess the normal distribution. Results for normally distributed data are presented as means ± SEMs. Comparisons between 2 groups were performed using independent-sample 2-tailed *t* tests, while comparisons among multiple groups were conducted using 1-way ANOVA followed by the least significant difference post hoc test. Repeated measures data were analyzed using 2-way ANOVA. For all statistical tests, *P* < 0.05 was considered significant. Statistical graphs were generated using GraphPad Prism 9.0 software.

### Study approval.

All experiments involving mice were approved by the Animal Ethics Committee of Anhui Medical University (Approval No. LLSC20211519).

### Data availability.

The RNA-Seq data used in this study are available in the National Center for Biotechnology Information Sequence Read Archive under the BioProject accession number PRJNA1246739. Values for all data points in graphs are reported in the Supporting Data Values File.

## Author contributions

YD, BS, SD, and ML designed the research. YD performed the major experiments. JZ contributed to the cellular experiments. JZ, CG, and WD contributed to the animal experiments. LF and HL contributed to the data analysis. YD wrote the manuscript. BS and SD revised the manuscript. SD, BS, and ML supervised the entire study. All authors read and approved the final manuscript.

## Supplementary Material

Supplemental data

Unedited blot and gel images

Supporting data values

## Version 1 05/08/2025

In-Press Preview

## Version 2 06/09/2025

Electronic publication
