# Supplementary material for: LRP1 regulates asthmatic airway smooth muscle proliferation through FGF2/ERK signaling
Source: JCI Insight. 2025 May 8;10(11):e185975. doi: 10.1172/jci.insight.185975 (PMC12220950; doi:10.1172/jci.insight.185975)
Supplement: Supplemental data [file jciinsight-10-185975-s021.pdf]

Supplemental Material

Supplemental figure legend for graphical abstract:

Schematic diagram illustrating the role and mechanism of LRP1 in airway smooth muscle (ASM) proliferation in asthma. The expression of LRP1 is significantly increased in ASM cells of mice with OVA-induced chronic asthma. Increased LRP1 promotes ASM proliferation by activating the FGF2/ERK signaling pathway, thereby exacerbating airway remodeling in asthma. On the other hand, increased LRP1 activates the transcription of *Mt1-mmp*, thereby promoting the proteolytic process of LRP1 and generating more LRP1-ICD. In turn, elevated LRP1-ICD reduces the protein levels of full-length LRP1 by promoting its degradation via the lysosomal pathway, thereby preventing further exacerbation of asthma caused by excessive increases in LRP1. Created with BioRender.com.

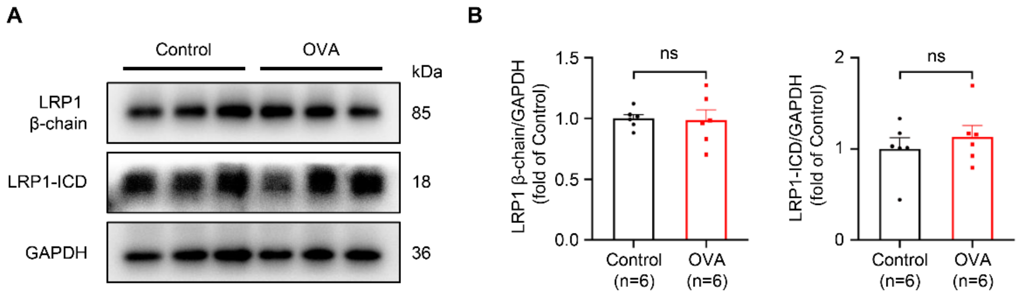

Supplemental Figure 1. (A-B) Representative immunoblot images (A) and summary data (B) showing LRP1 protein levels in lung tissues of control mice (Control) and mice treated with OVA to induce a chronic asthma model (OVA). n = 6 mice per group. All data were analyzed using independent samples t-tests and are presented as means ± SEMs. ns, no statistical significance.

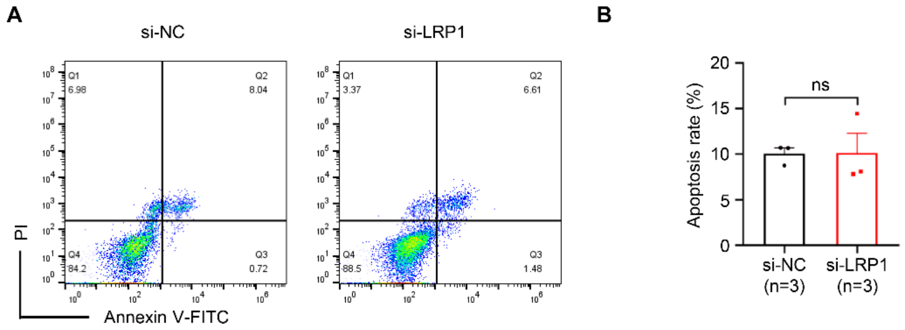

Supplemental Figure 2. (A-B) Representative scatter plots (A) and summary data (B) showing the proportion of apoptotic cells in human bronchial smooth muscle cells transfected with *LRP1*-targeting siRNA (si-LRP1) or negative control siRNA (si-NC) (n = 3). All data were analyzed using independent samples t-tests and are presented as means ± SEMs. ns, no statistical significance.

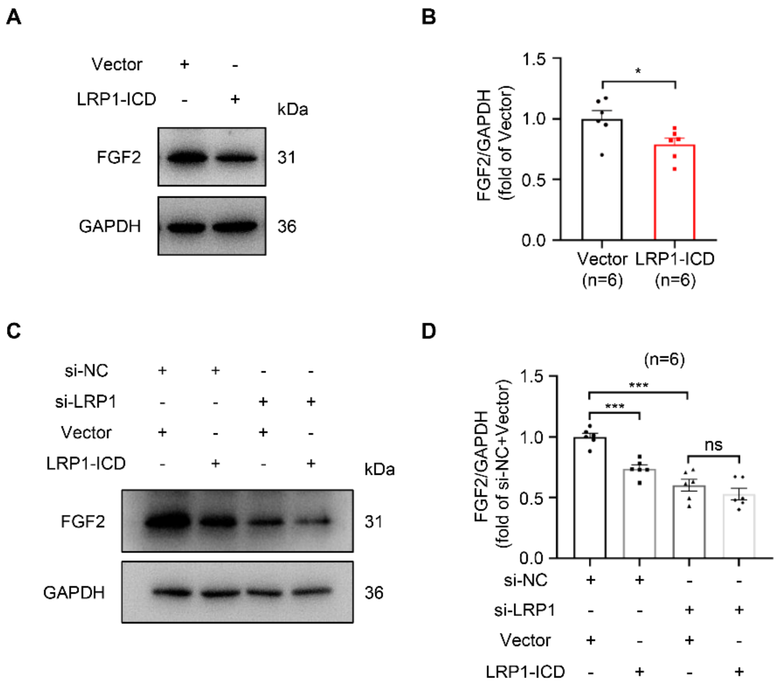

Supplemental Figure 3. (A-B) Human bronchial smooth muscle cells (HBSMCs) were transfected with recombinant plasmids overexpressing LRP1-ICD (LRP1-ICD) or empty vector (Vector). Representative immunoblot images (A) and summary data (B) showing FGF2 protein levels (n = 6). (C-D) HBSMCs were transfected with *LRP1*-targeting siRNA (si-LRP1) or negative control siRNA (si-NC), along with recombinant plasmids overexpressing LRP1-ICD (LRP1-ICD) or empty vector (Vector). Representative immunoblot images showing FGF2 protein levels (n = 6). All data were analyzed using independent samples t-tests or one-way ANOVA and are presented as means ± SEMs. ns, no statistical significance, *p<0.05, ***p<0.001.

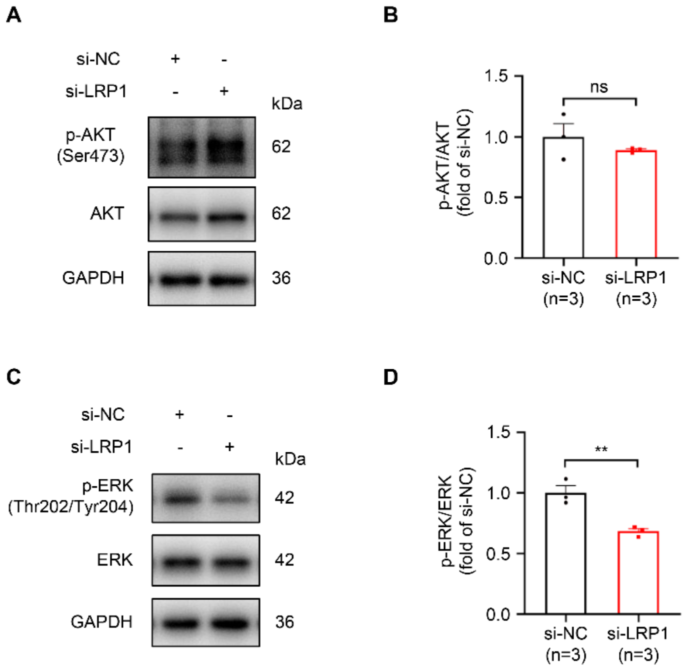

Supplemental Figure 4. (A-B) HBSMCs were transfected with *LRP1*-targeting siRNA (si-LRP1) or negative control siRNA (si-NC). Representative immunoblot images (A) and summary data (B) showing the protein levels of phosphorylated (p)-AKT (Ser473) and total AKT in indicated cells (n = 3). (C-D) Representative immunoblot images (C) and summary data (D) showing the protein levels of p-ERK (Thr202/Tyr204) and total ERK in indicated cells (n = 3). All data were analyzed using independent samples t-tests and are presented as means ± SEMs. ns, no statistical significance, **p<0.01.

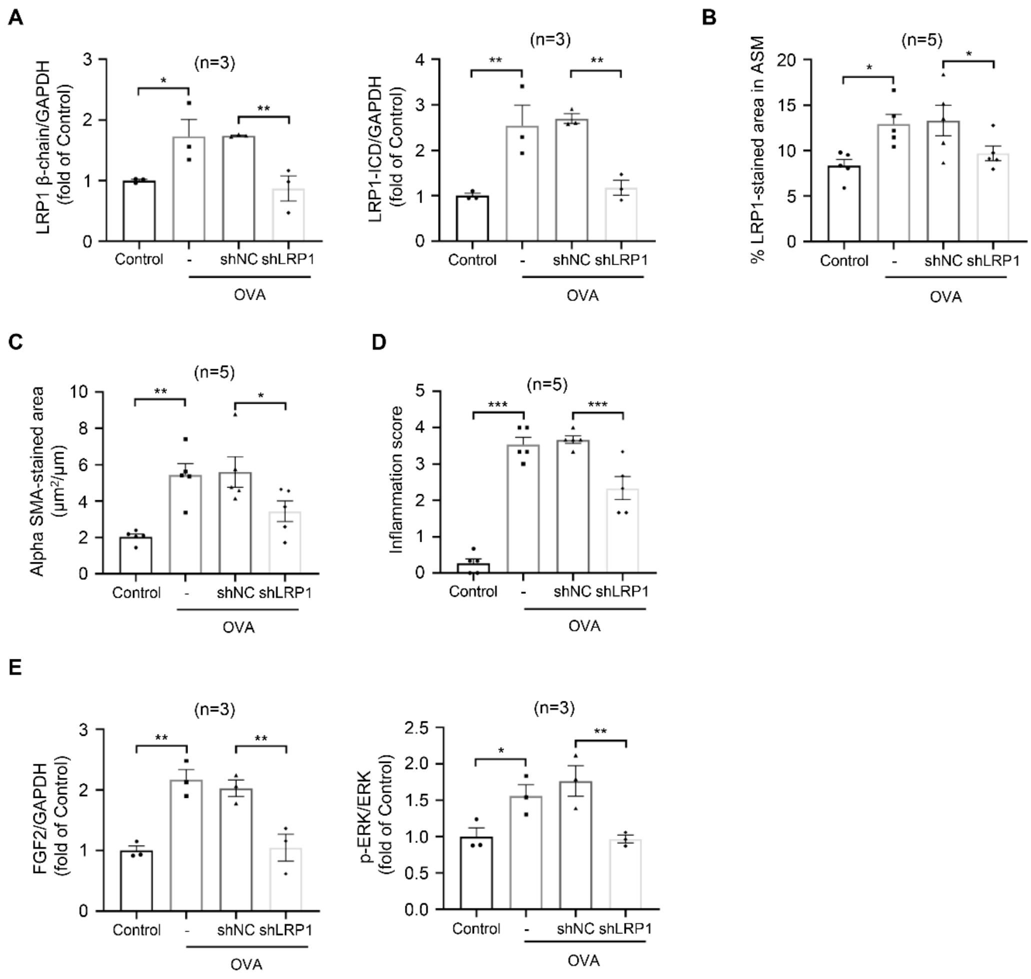

Supplemental Figure 5. (A) Mice were assigned to one of four groups: Control, OVA-induced chronic asthma (OVA), OVA administration concurrent with intratracheal instillation of lentivirus containing negative control shRNA (OVA+shNC), and OVA treatment concurrent with intratracheal instillation of lentivirus containing LRP1-targeting shRNA (OVA+shLRP1). Summary data showing protein levels of LRP1 β-chain and LRP1-ICD in tracheal tissues of the four groups of mice. n = 3 mice per group. (B) Summary data showing LRP1 protein levels in airway smooth muscle (ASM) of the four groups of mice. n = 5 mice per group. (C) Summary data showing alpha alpha smooth muscle actin (SMA) protein levels in lung tissues of the four groups of mice. n = 5 mice per group. (D) Summary data showing inflammation scores for lung tissues in the indicated mice. n = 5 mice per group. (E) Summary data showing protein levels of FGF2 and the ratio of phosphorylated (p)-ERK (Thr202/Tyr204) to total ERK levels in tracheal tissues of the four groups of mice. n = 3 mice per group. All data were analyzed using one-way ANOVA and are presented as means ± SEMs. *p<0.05, **p<0.01, ***p<0.001.

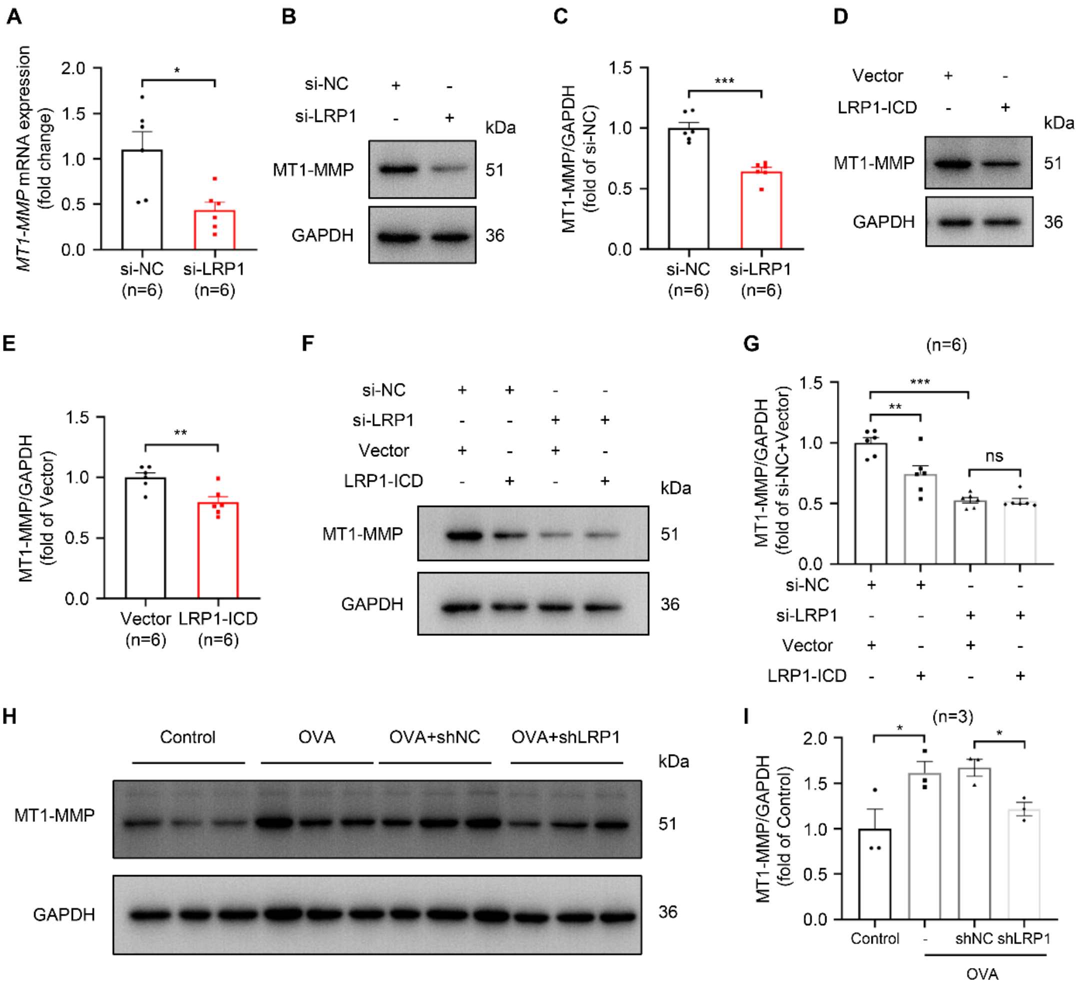

Supplemental Figure 6. (A) Human bronchial smooth muscle cell (HBSMC) was transfected with *LRP1*-targeting siRNA (si-LRP1) or negative control siRNA (si-NC). Summary data showing the relative expression levels of *MT1-MMP* mRNA in indicated cells. *GAPDH* was used as an internal reference (n = 6). (B-C) Representative immunoblot images (B) and summary data (C) showing MT1-MMP protein levels in indicated cells (n = 6). (D-E) HBSMCs were transfected with recombinant plasmids overexpressing LRP1-ICD (LRP1-ICD) or empty vector (Vector). Representative immunoblot images (D) and summary data (E) showing MT1-MMP protein levels (n = 6). (F-G) HBSMCs were transfected with *LRP1*-targeting siRNA (si-LRP1) or negative control siRNA (si-NC), along with recombinant plasmids overexpressing LRP1-ICD (LRP1-ICD) or empty vector (Vector). Representative immunoblot images (F) and summary data (G) showing MT1-MMP protein levels (n = 6). (H-I) Mice were assigned to one of four groups: Control, OVA-induced chronic asthma (OVA), OVA treatment concurrent with intratracheal instillation of lentivirus containing negative control shRNA (OVA+shNC), and OVA treatment concurrent with intratracheal instillation of lentivirus containing *Lrp1*-targeting shRNA

(OVA+shLRP1). Representative immunoblot images showing MT1-MMP protein levels in tracheal tissues from the four groups of mice. n = 3 mice per group. All data were analyzed using independent samples t-tests or one-way ANOVA and are presented as means ± SEMs. ns, no statistical significance, *p<0.05, **p<0.01, ***p<0.001.

Supplemental Table 1. Primer sequences applied for gene expression analysis in human bronchial smooth muscle cells and tracheal tissues.

Gene	Forward primer 5′-3′	Reverse primer 5′-3′
ms-Lrp1	CCACTATGGATGCCCCTAAAAC	GCAATCTCTTTCACCGTCACA
ms-Mt1-mmp	ACCCACACACAACGCTCA	GCCTGTCACTTGTAAACCATAGA
ms-Adam17	ACCACTTTGGTGCCTTTCGT	GTCGCAGACTGTAGATCCCTT
ms-Psenen	ATGAACTTGGAGCGGGTATCC	CGAGGAACGCCTCTCTGAAG
ms-Gapdh	AGGTCGGTGTGAACGGATTTG	TGTAGACCATGTAGTTGAGGTCA
hs-LRP1	TCTACTTTGCCGACACCACC	TGTCTTTTTGGGCCCATCGT
hs-FGF2	GCGACCCTCACATCAAGCTA	CCATCTTCCTTCATAGCCAGGT
hs-MT-MMP	GAGCATTCCAGTGACCCCTC	ACCCTGACTCACCCCCATAA
hs-GAPDH	CAGGAGGCATTGCTGATGAT	GAAGGCTGGGGCTCATTT
