# Supplementary material for: LRP1 regulates asthmatic airway smooth muscle proliferation through FGF2/ERK signaling
Source: JCI Insight. 2025 May 8;10(11):e185975. doi: 10.1172/jci.insight.185975 (PMC12220950; doi:10.1172/jci.insight.185975)

Unedited blot and gel images

Full unedited gel for **Figure 1G**

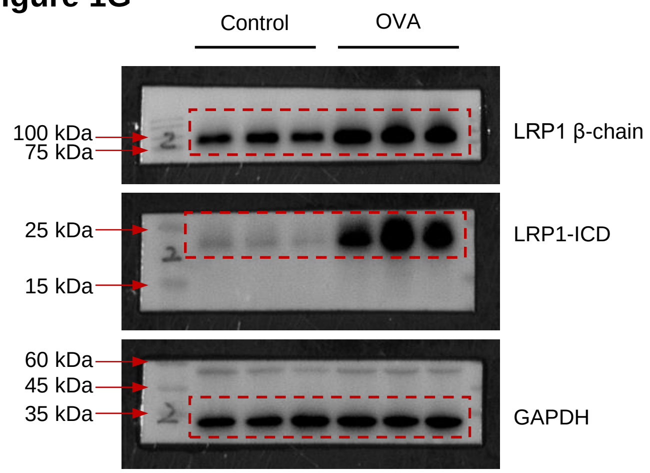

Full unedited gel for **Figure 2A**

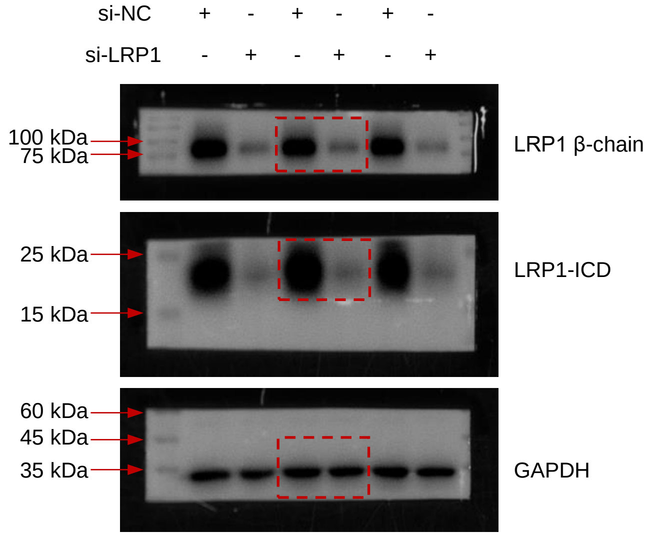

Full unedited gel for **Figure 3A**

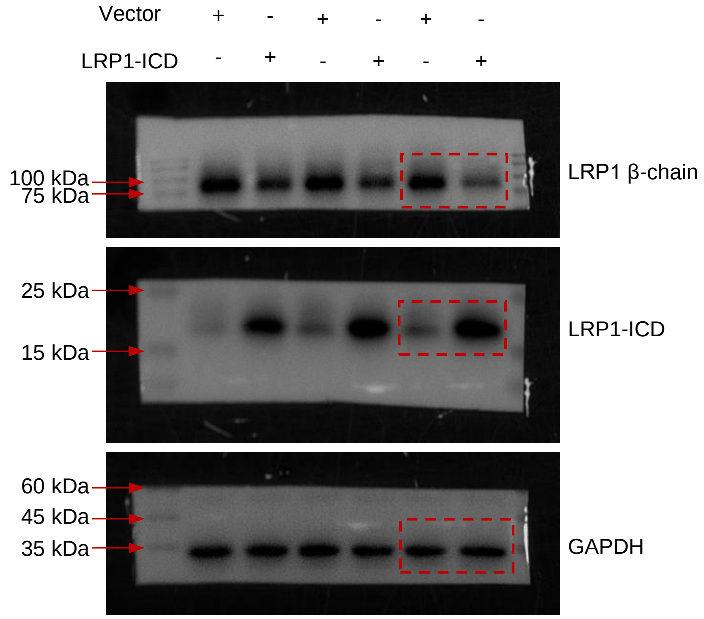

Full unedited gel for **Figure 3C**

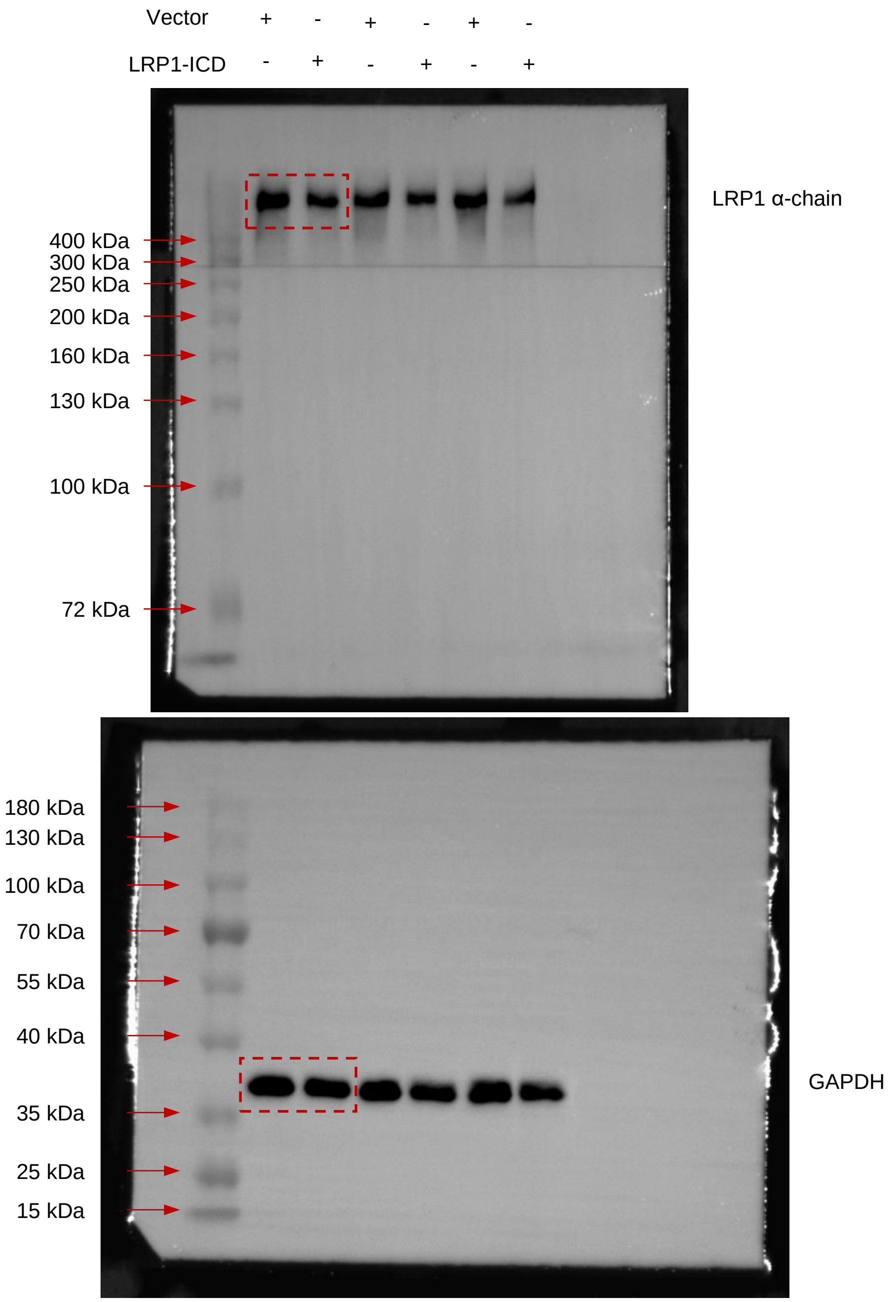

Full unedited gel for **Figure 4A**

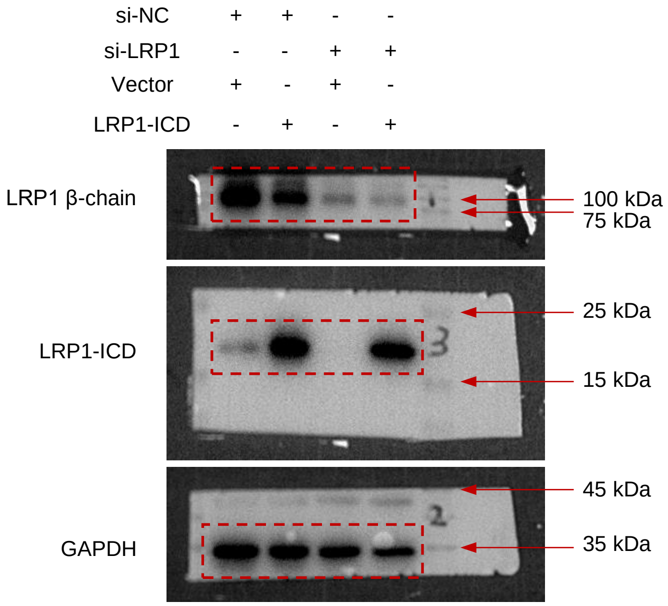

Full unedited gel for **Figure 5D**

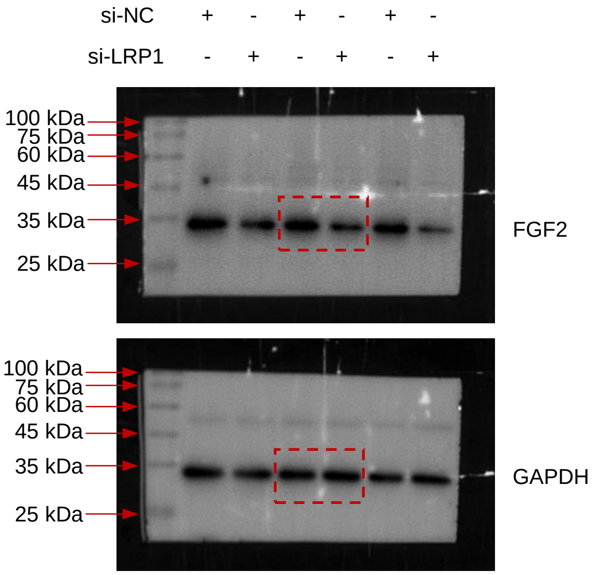

Full unedited gel for **Figure 5F**

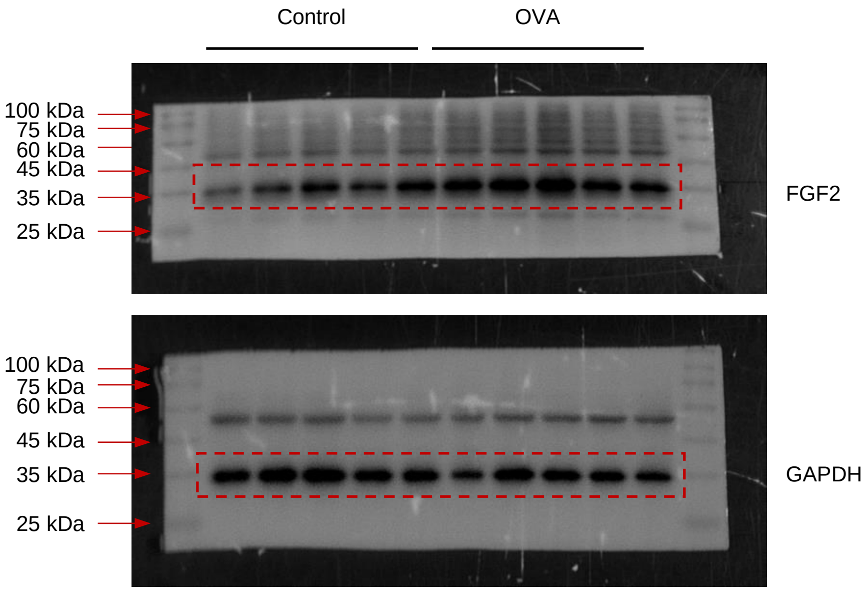

Full unedited gel for **Figure 7C**

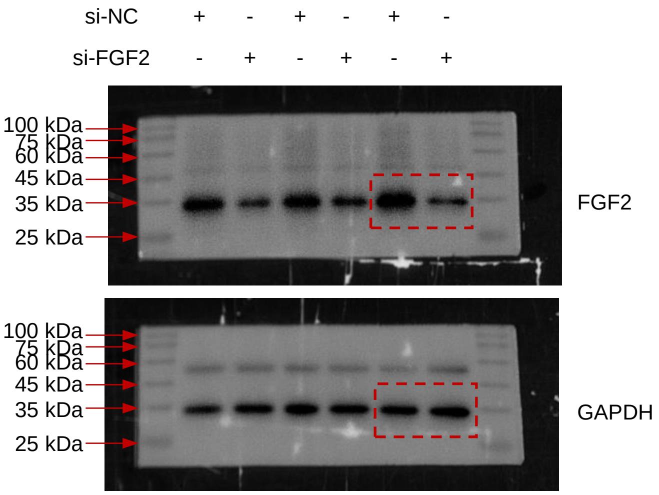

Full unedited gel for **Figure 7E**

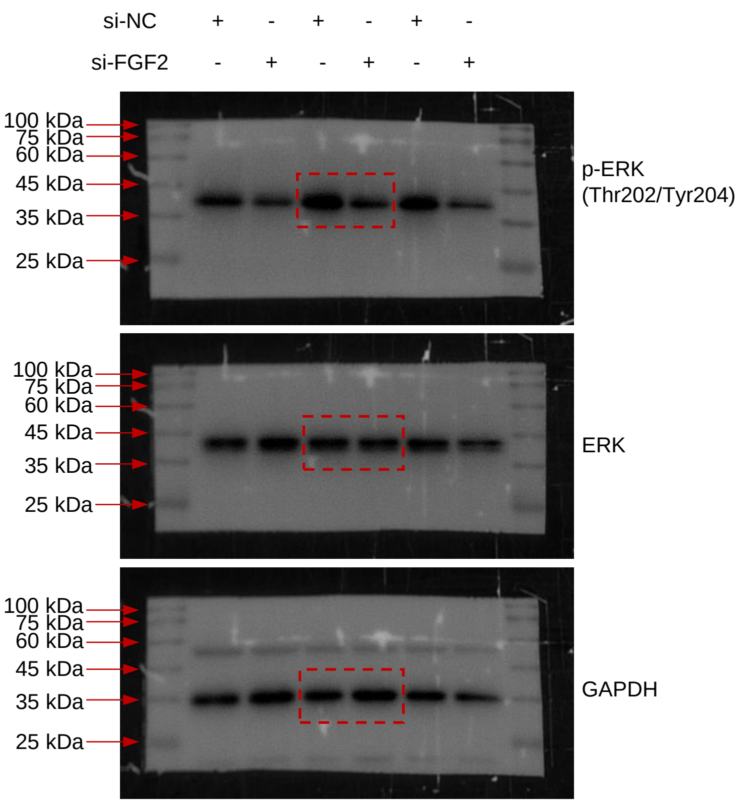

Full unedited gel for **Figure 7G**

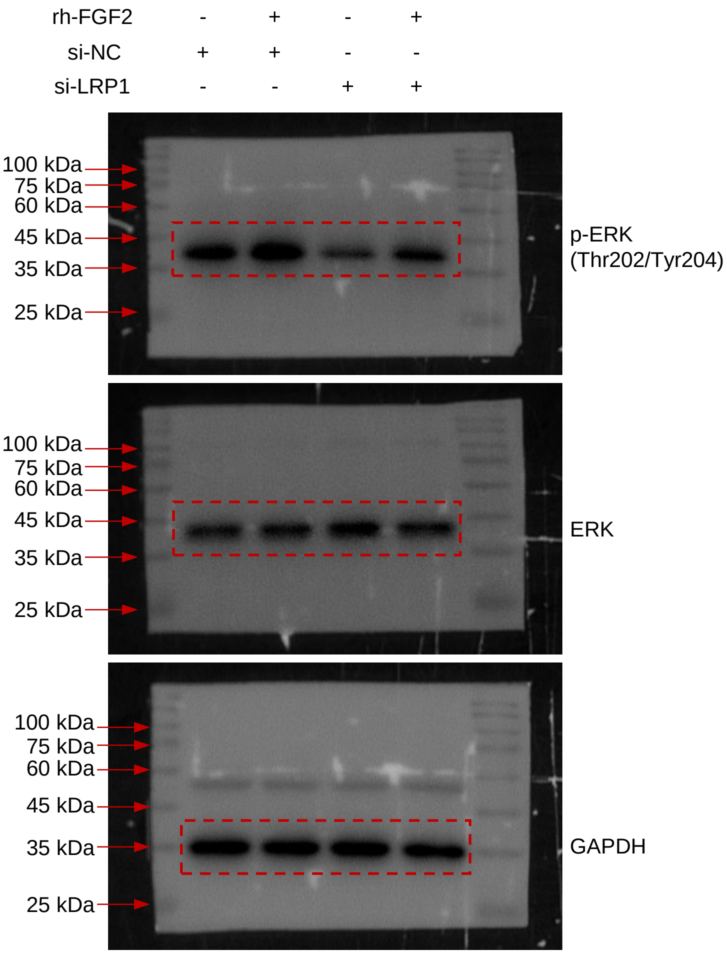

Full unedited gel for **Figure 8B**

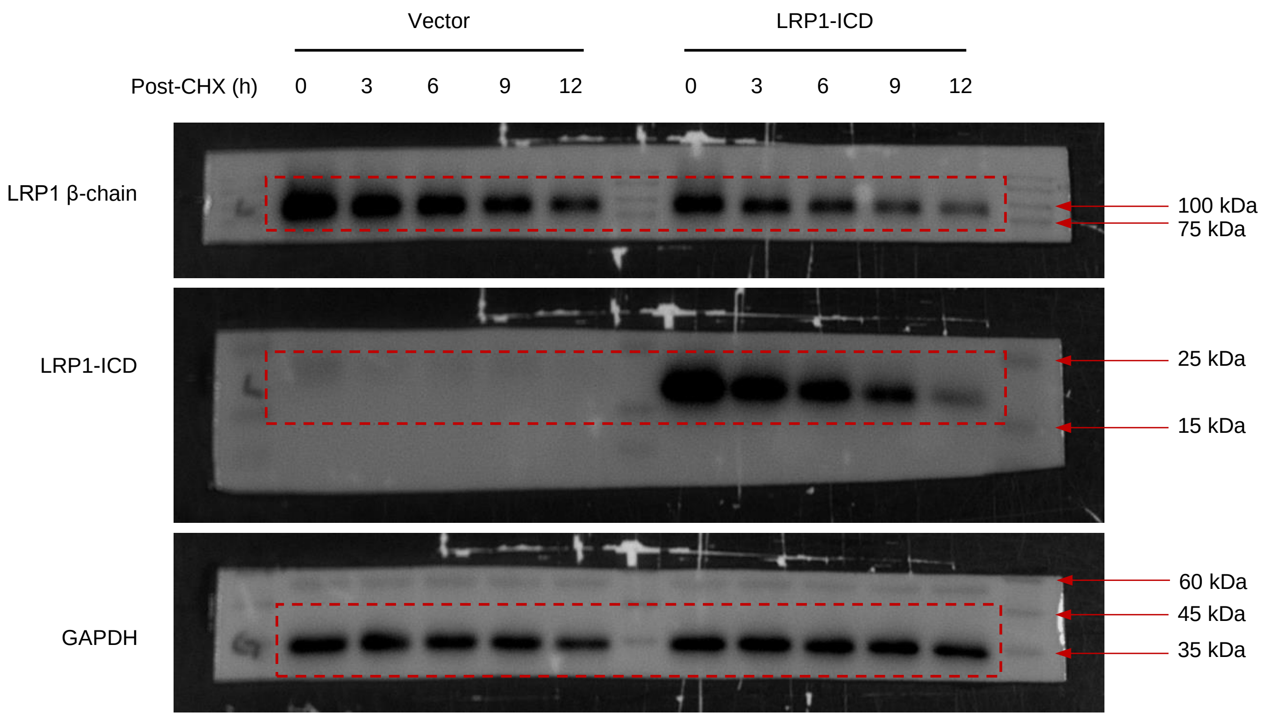

Full unedited gel for **Figure 8D**

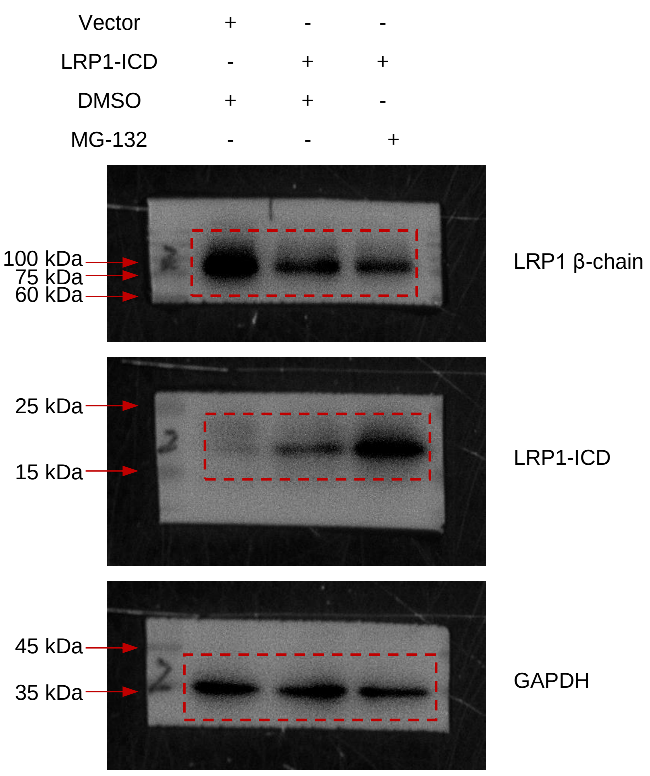

Full unedited gel for **Figure 8F**

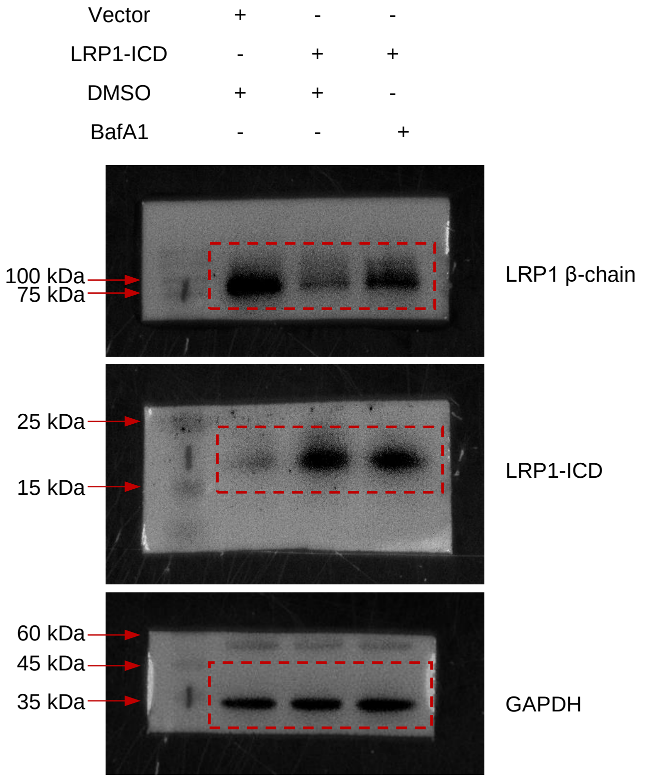

Full unedited gel for **Figure 9B**

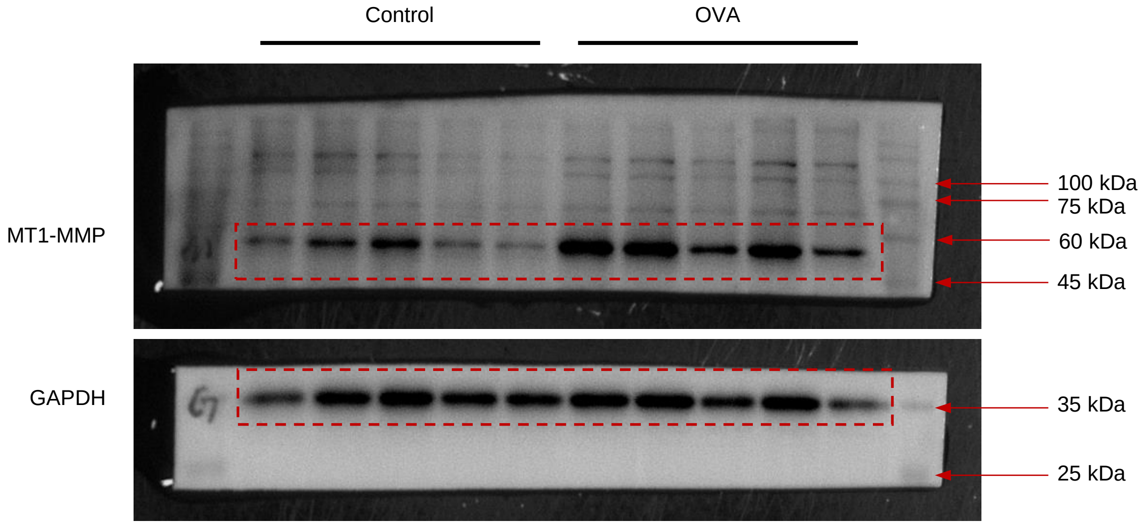

Full unedited gel for **Figure 9D**

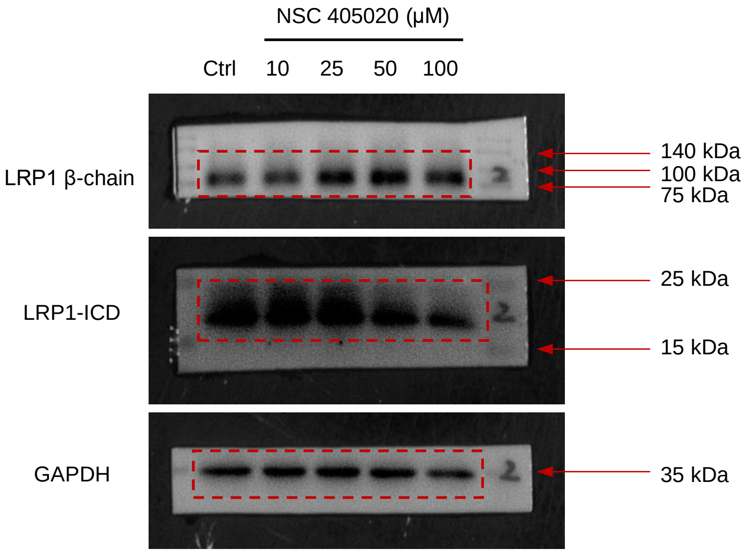

Full unedited gel for **Figure 10A**

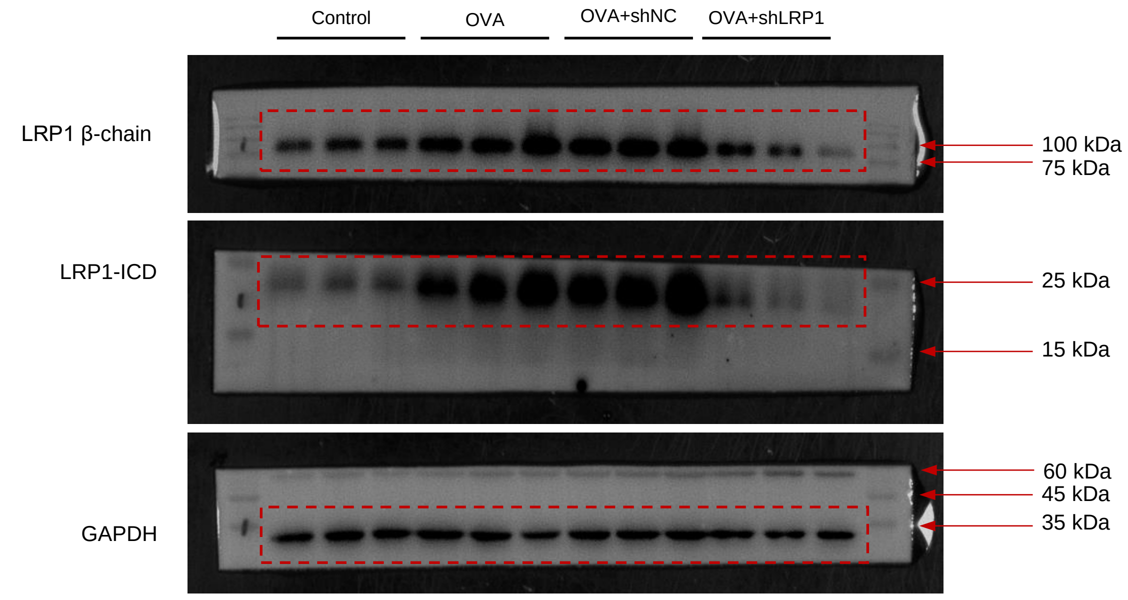

Full unedited gel for **Figure 10G**

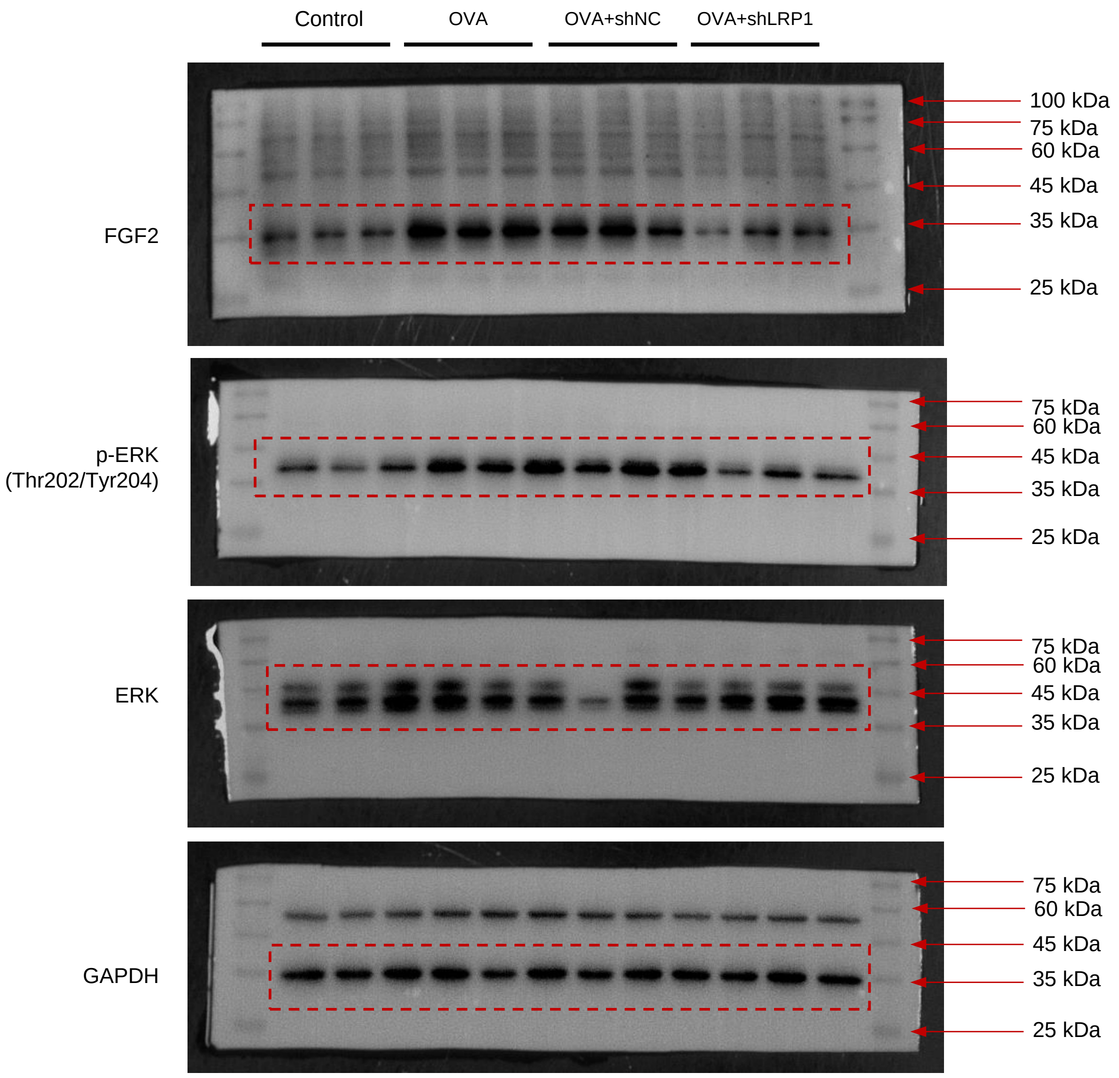

Full unedited gel for **Supplemental Figure 1A**

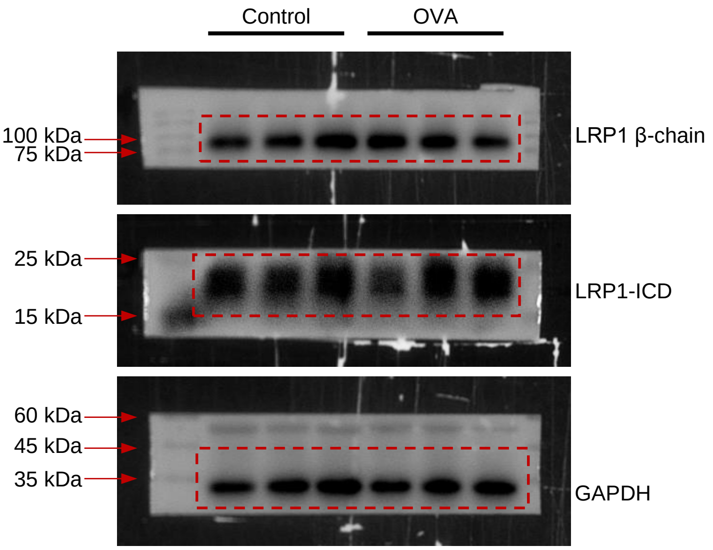

Full unedited gel for **Supplemental Figure 3A**

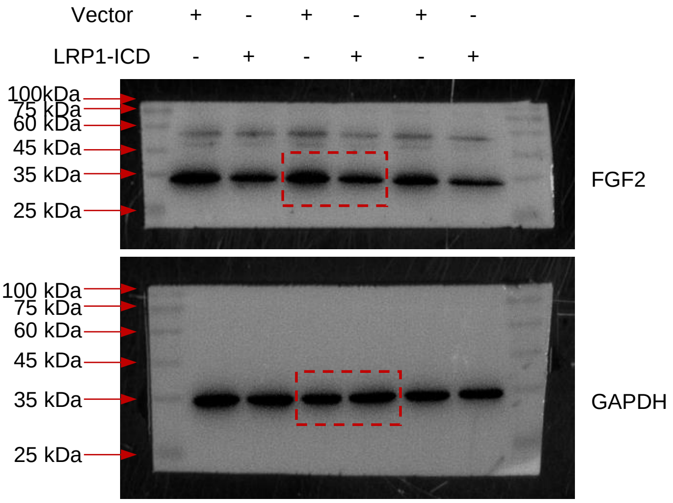

Full unedited gel for **Supplemental Figure 3C**

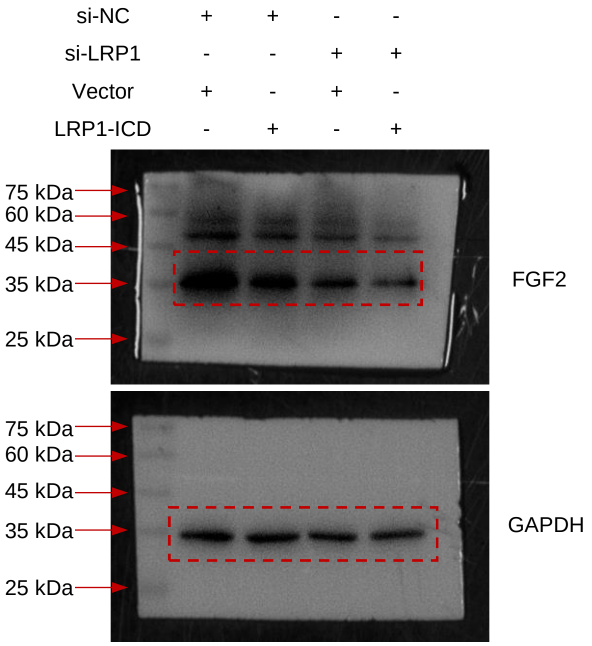

Full unedited gel for **Supplemental Figure 4A**

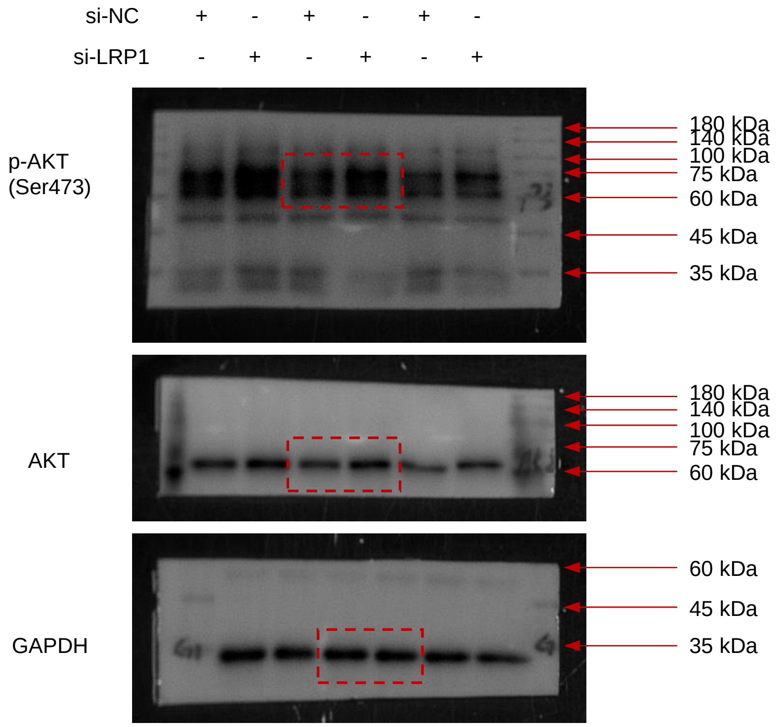

Full unedited gel for **Supplemental Figure 4C**

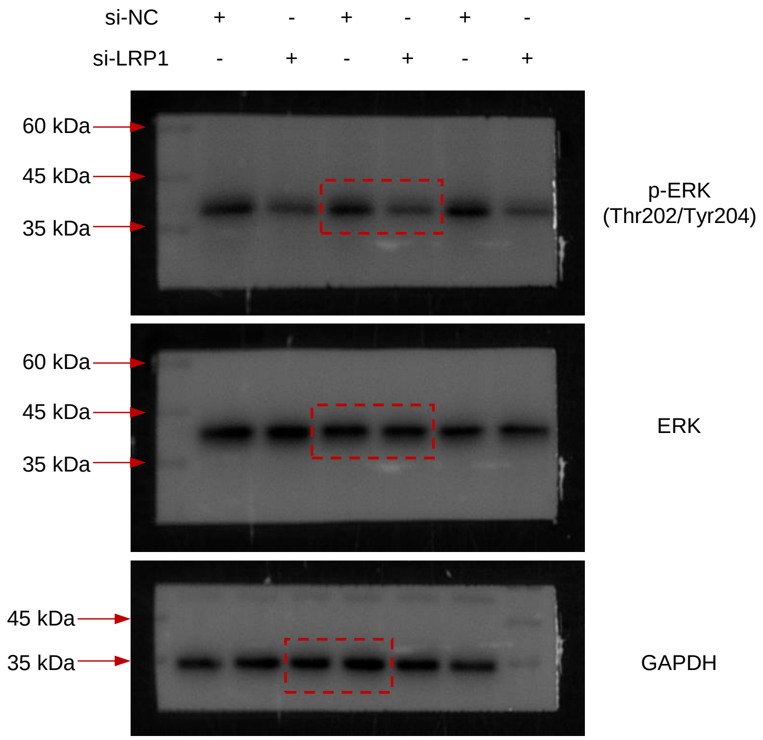

Full unedited gel for **Supplemental Figure 6B**

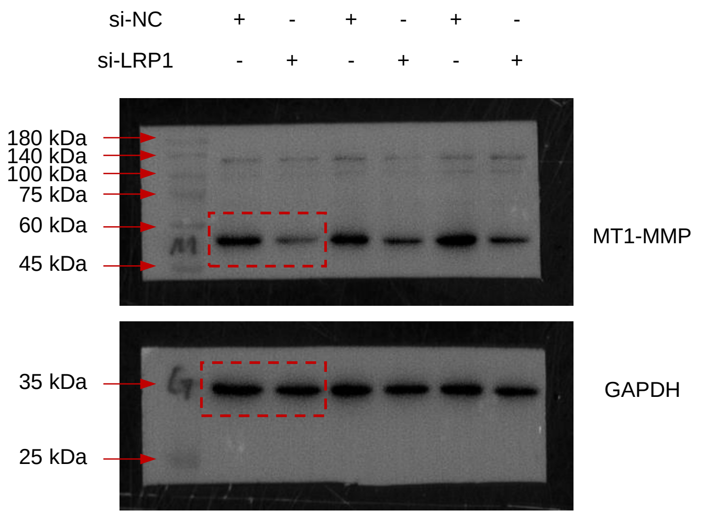

Full unedited gel for **Supplemental Figure 6D**

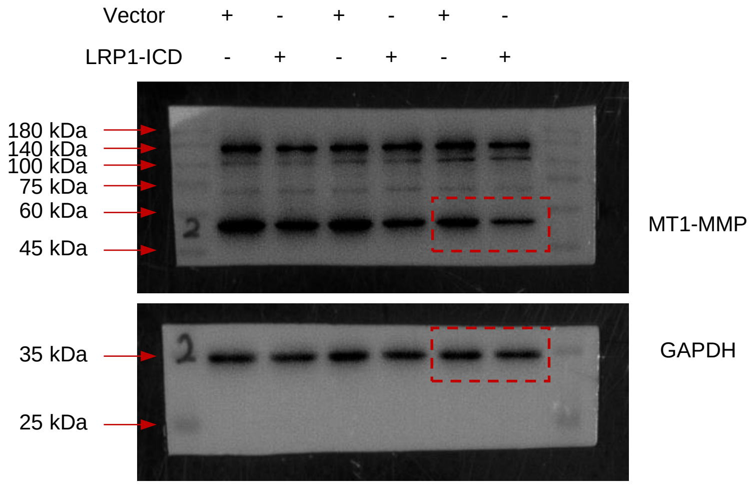

Full unedited gel for **Supplemental Figure 6F**

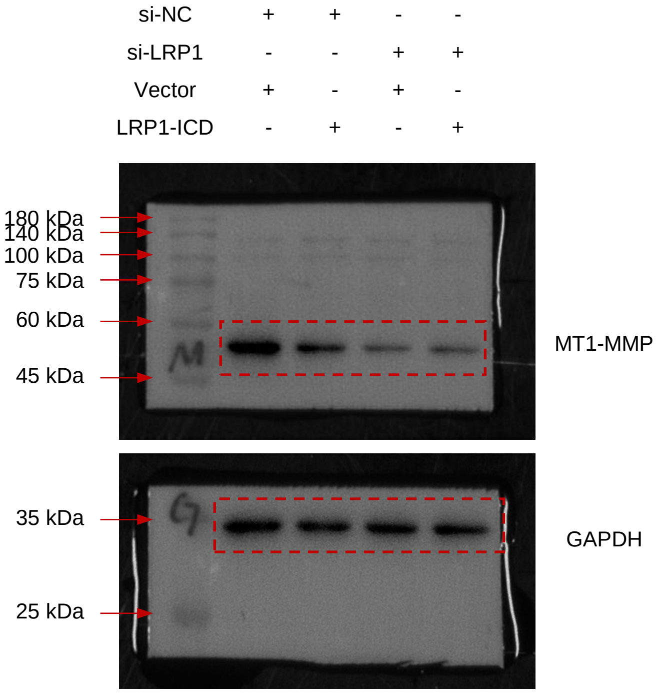

Full unedited gel for **Supplemental Figure 6H**

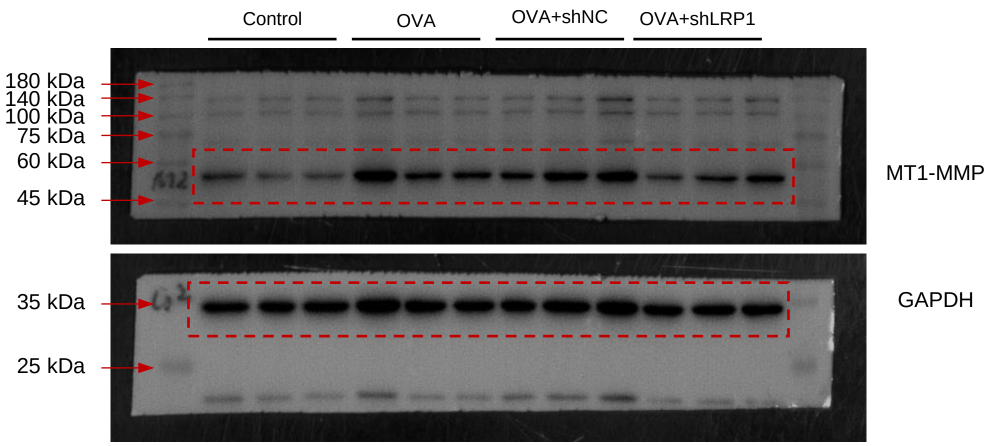

Supplement: Unedited blot and gel images [file jciinsight-10-185975-s022.pdf]
